# Supplementary material for: Cardiac differentiation of human pluripotent stem cells using defined extracellular matrix proteins reveals essential role of fibronectin
Source: eLife. 2022 Jun 27;11:e69028. doi: 10.7554/eLife.69028 (PMC9236614; doi:10.7554/eLife.69028)
Supplement: Figure 2—source data 1. [file elife-69028-fig2-data1.zip › Fig2A 20200706 for ImageJ.pptx]

## Slide 1
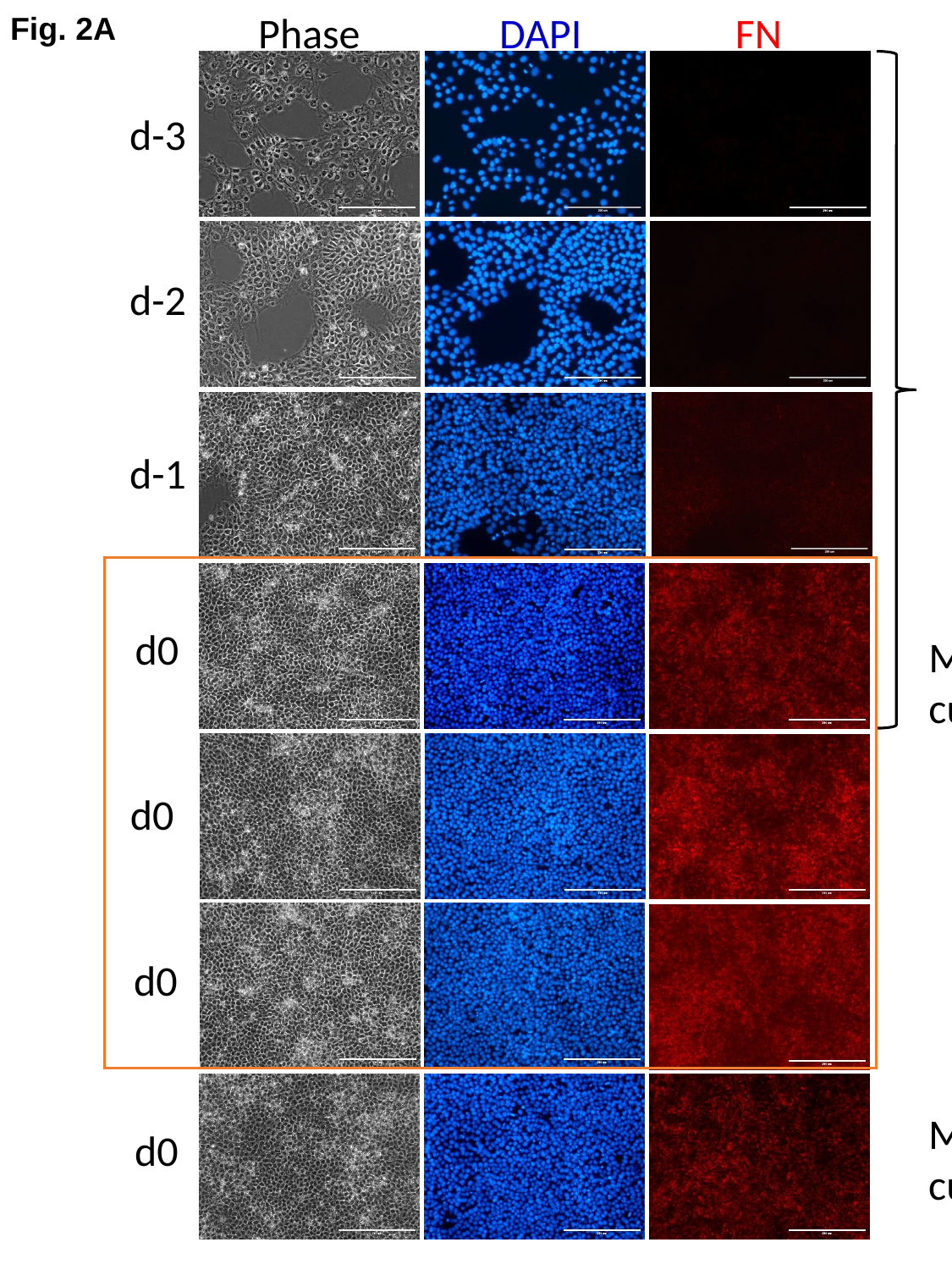

Phase
 DAPI
 FN
Fig. 2A
d-3
d-2
d-1
d0
Matrix sandwich culture
d0
d0
Monolayer culture
d0

## Slide 2
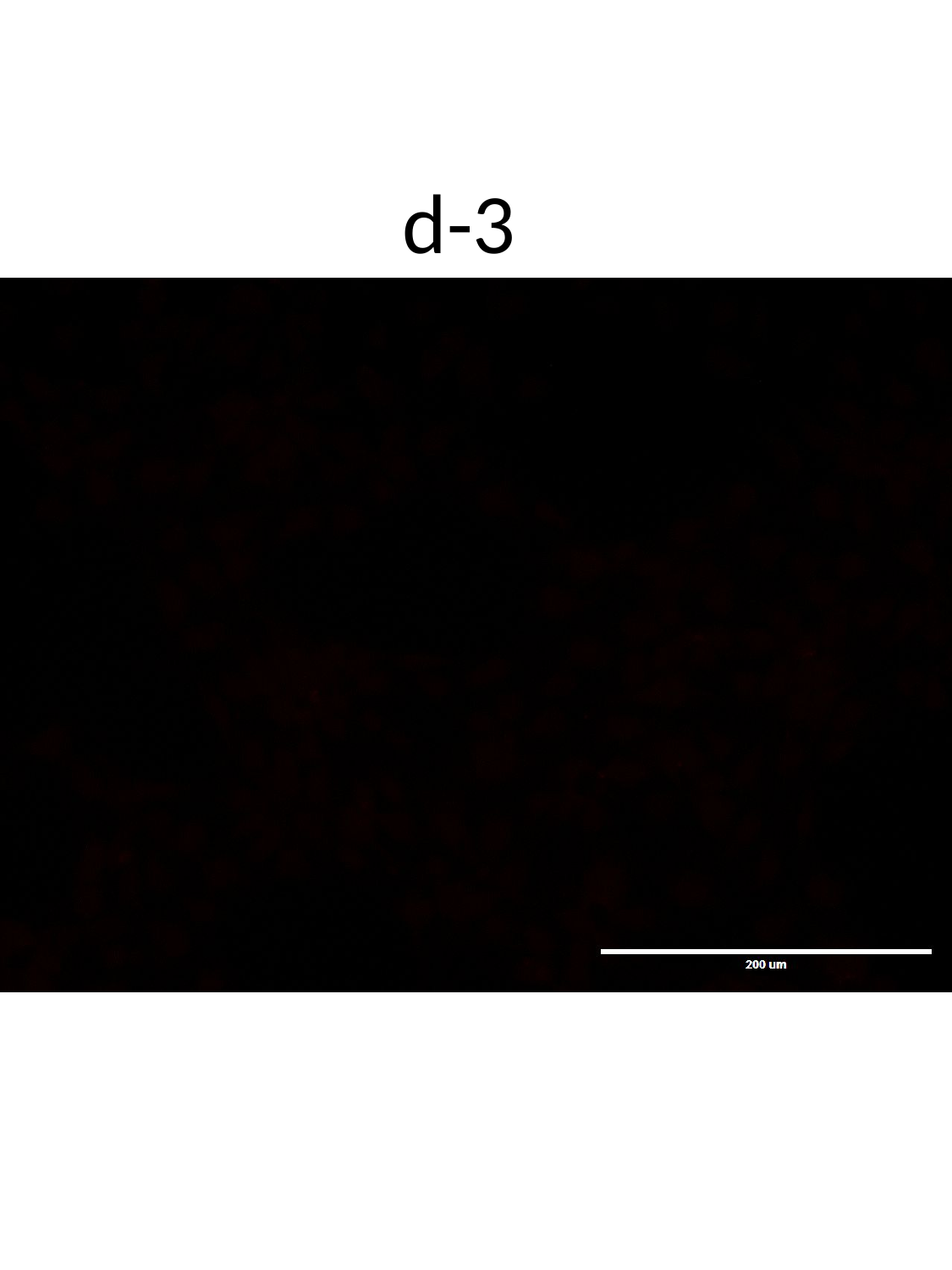

d-3

## Slide 3
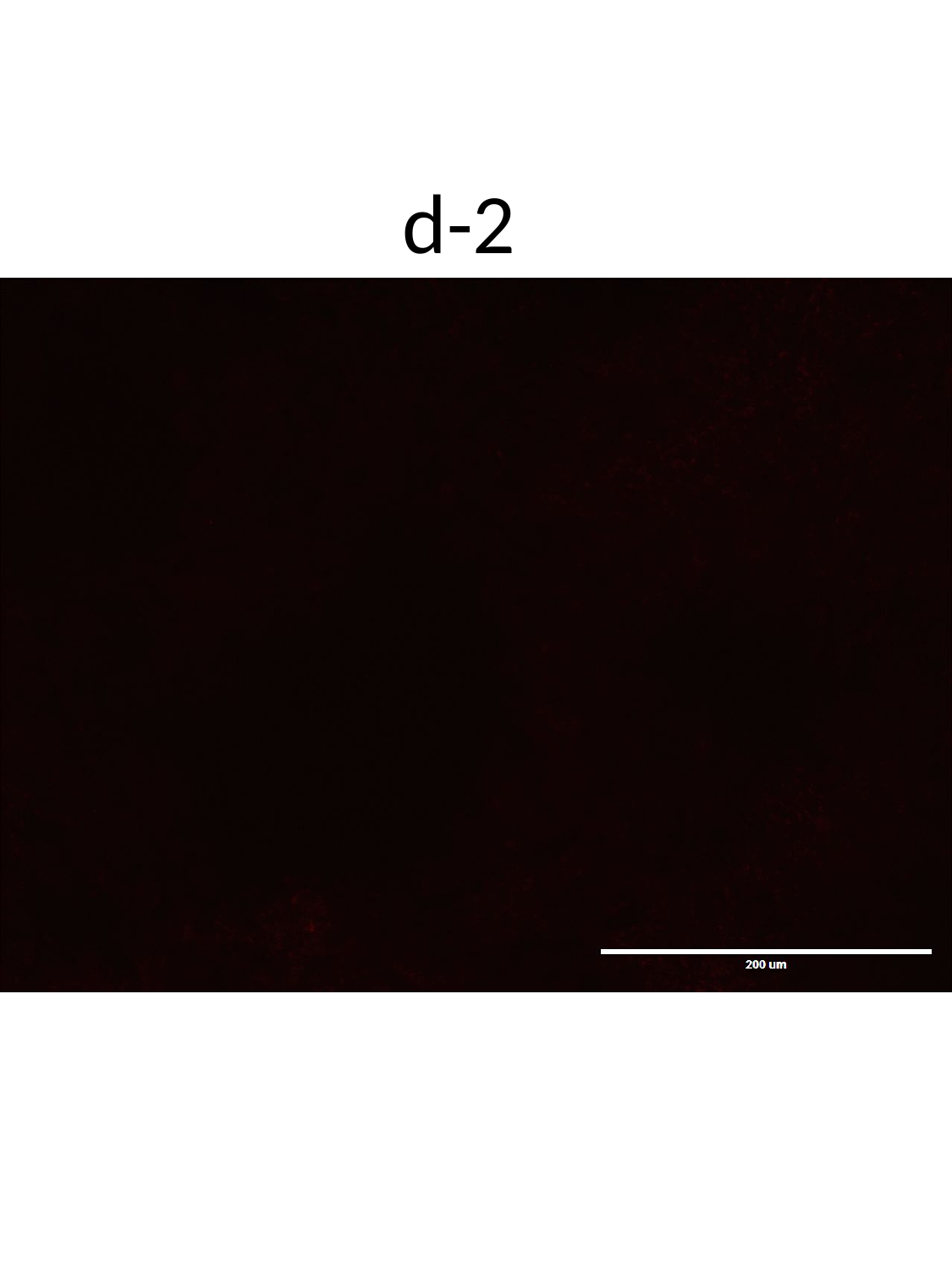

d-2

## Slide 4
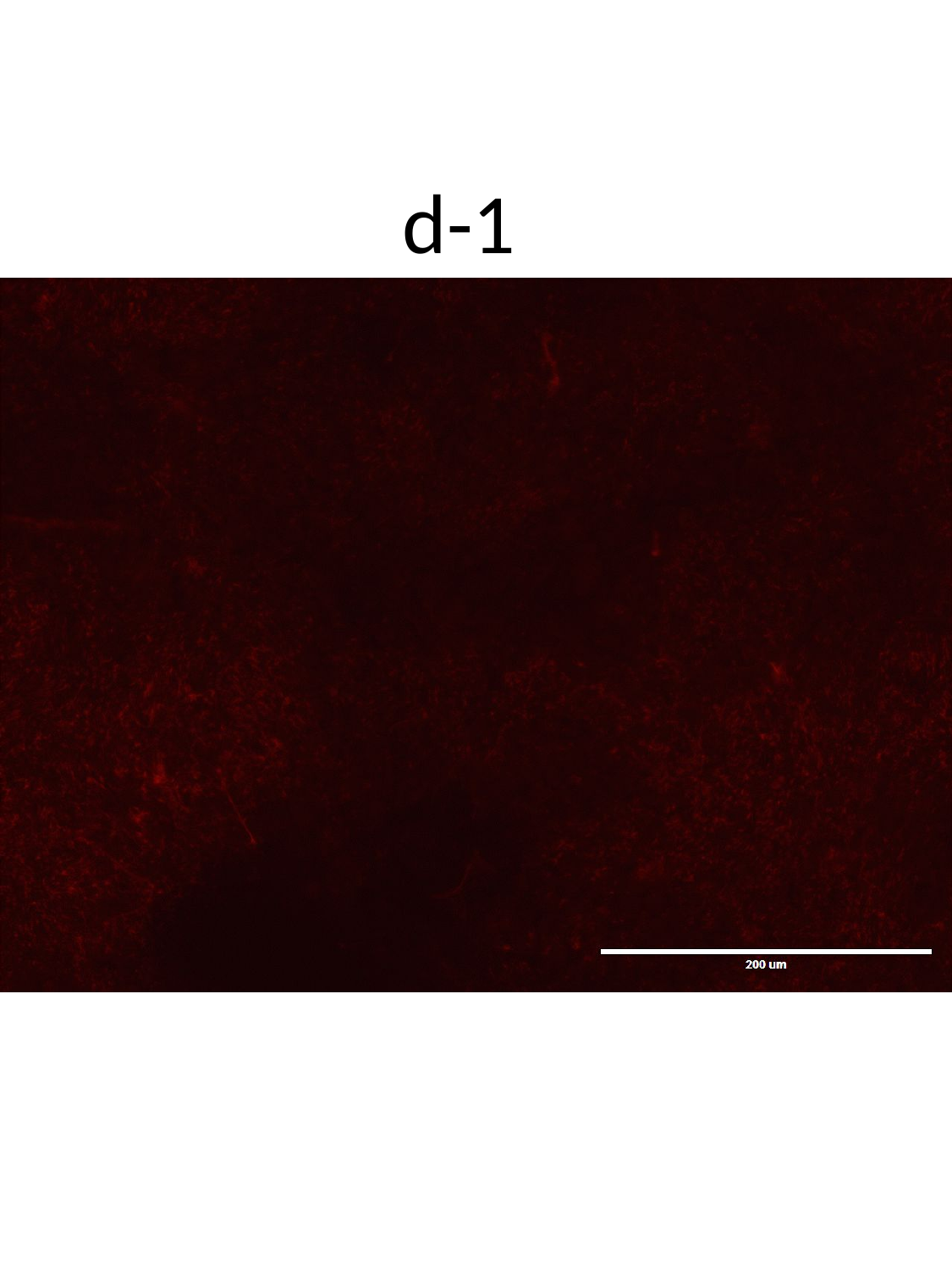

d-1

## Slide 5
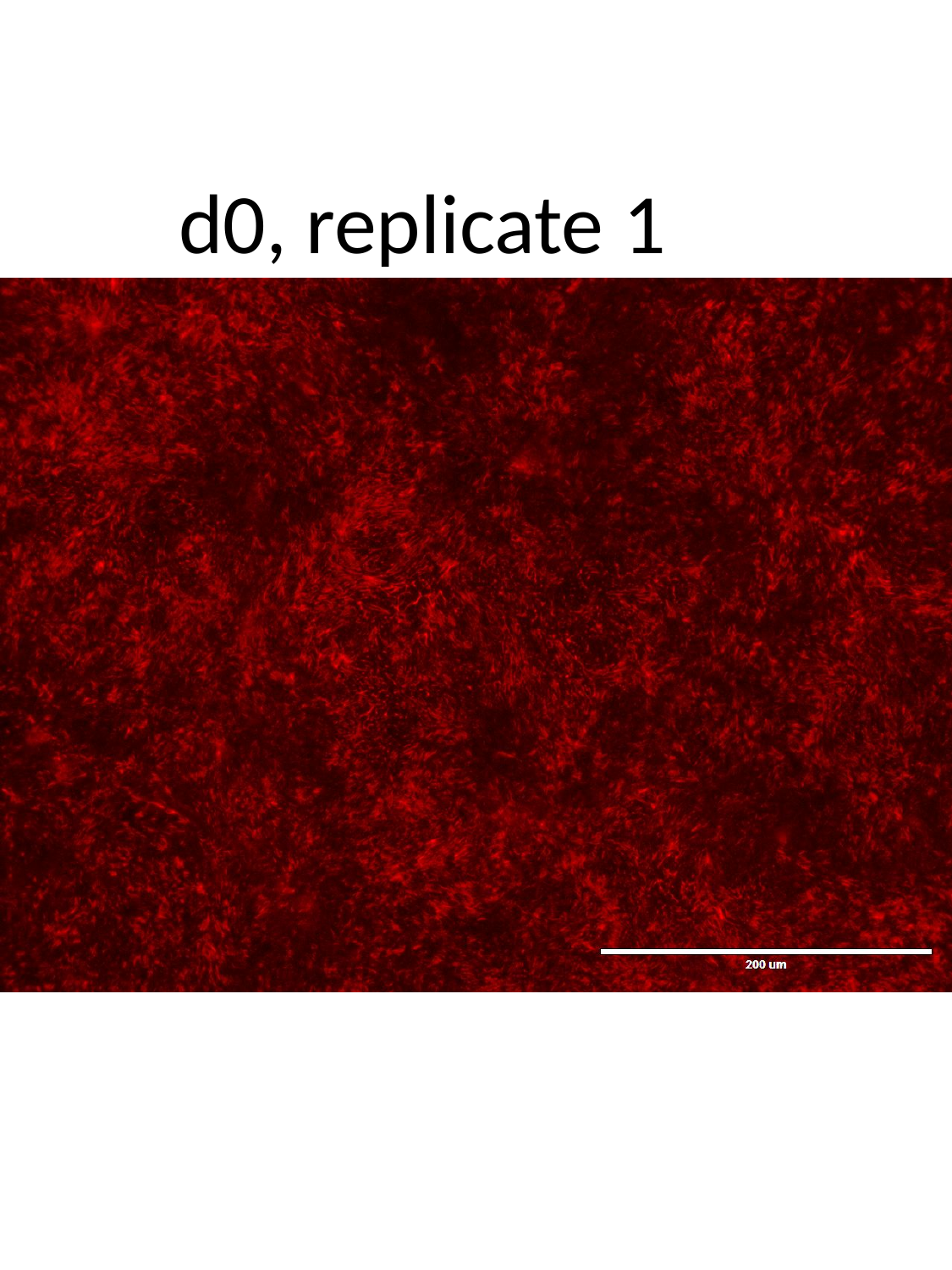

d0, replicate 1

## Slide 6
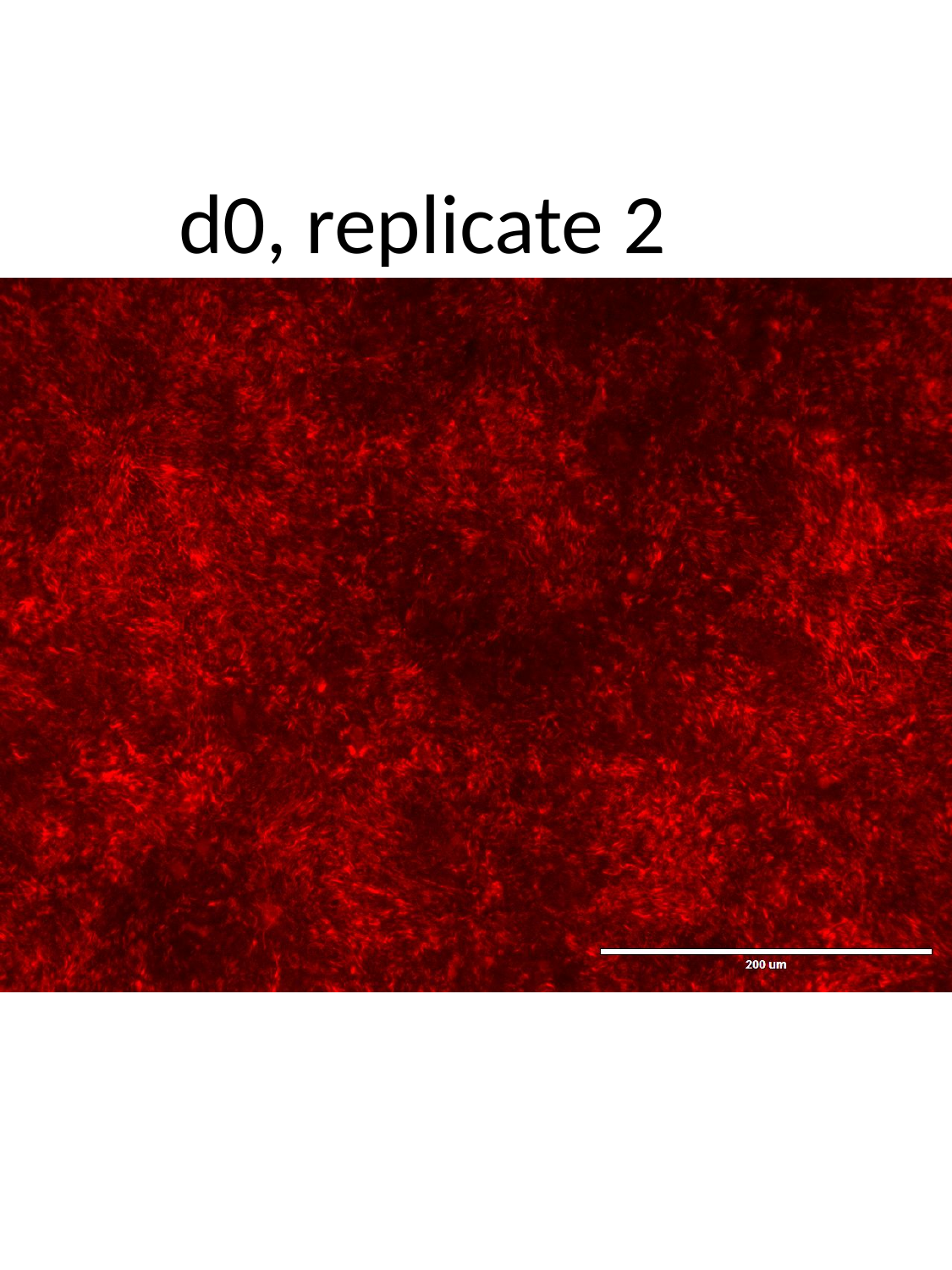

d0, replicate 2

## Slide 7
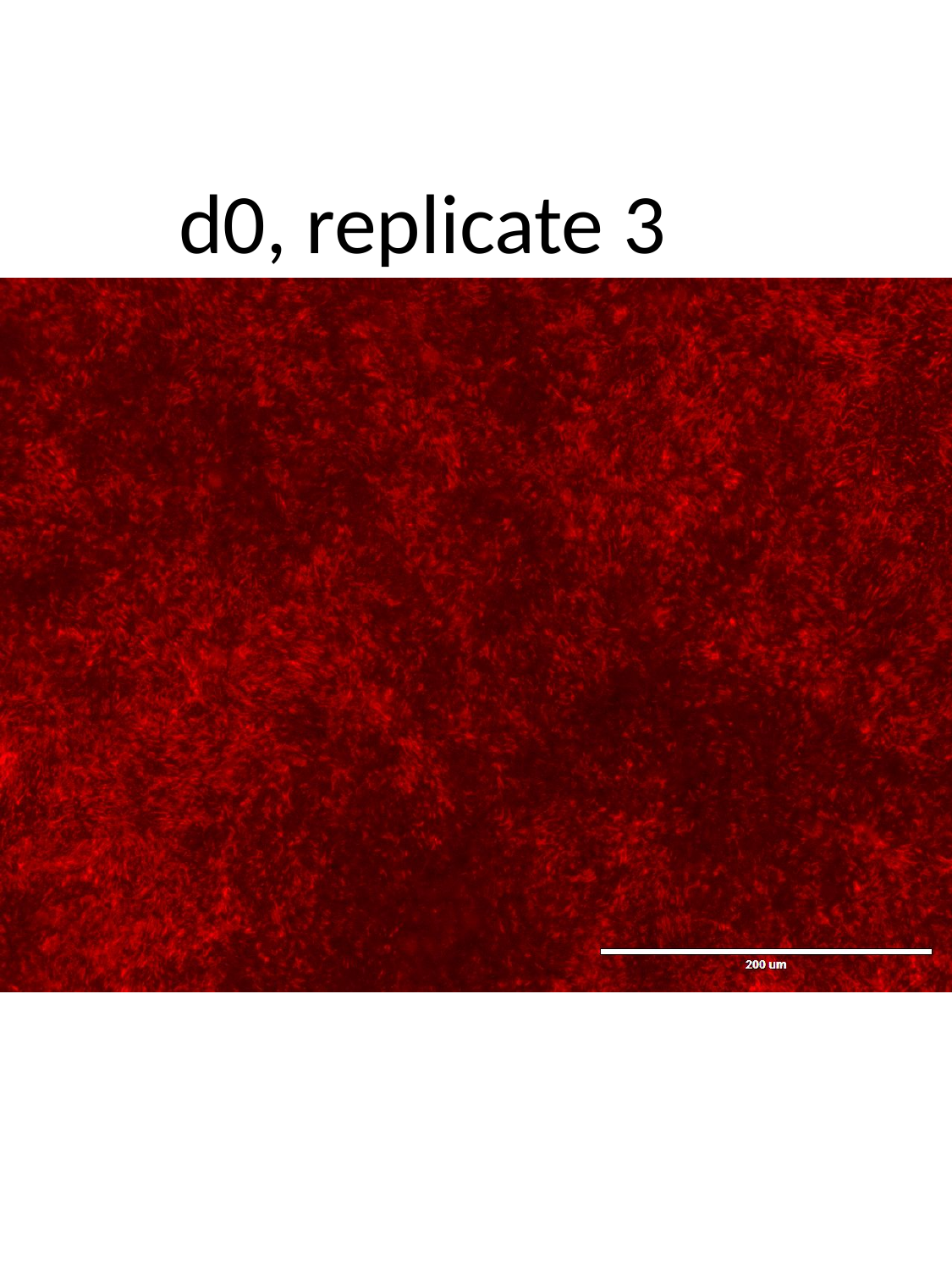

d0, replicate 3

## Slide 8
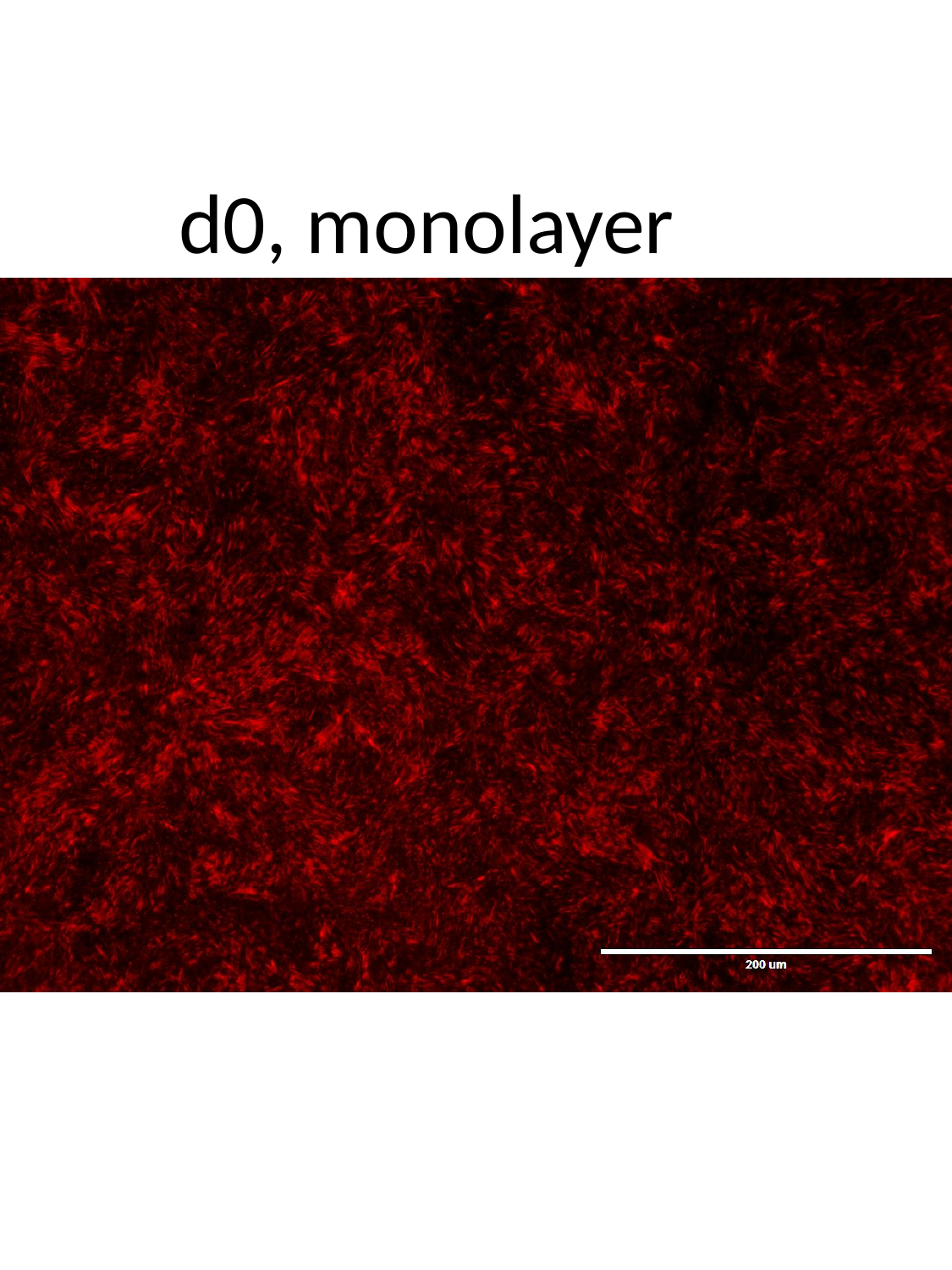

d0, monolayer

## Slide 9
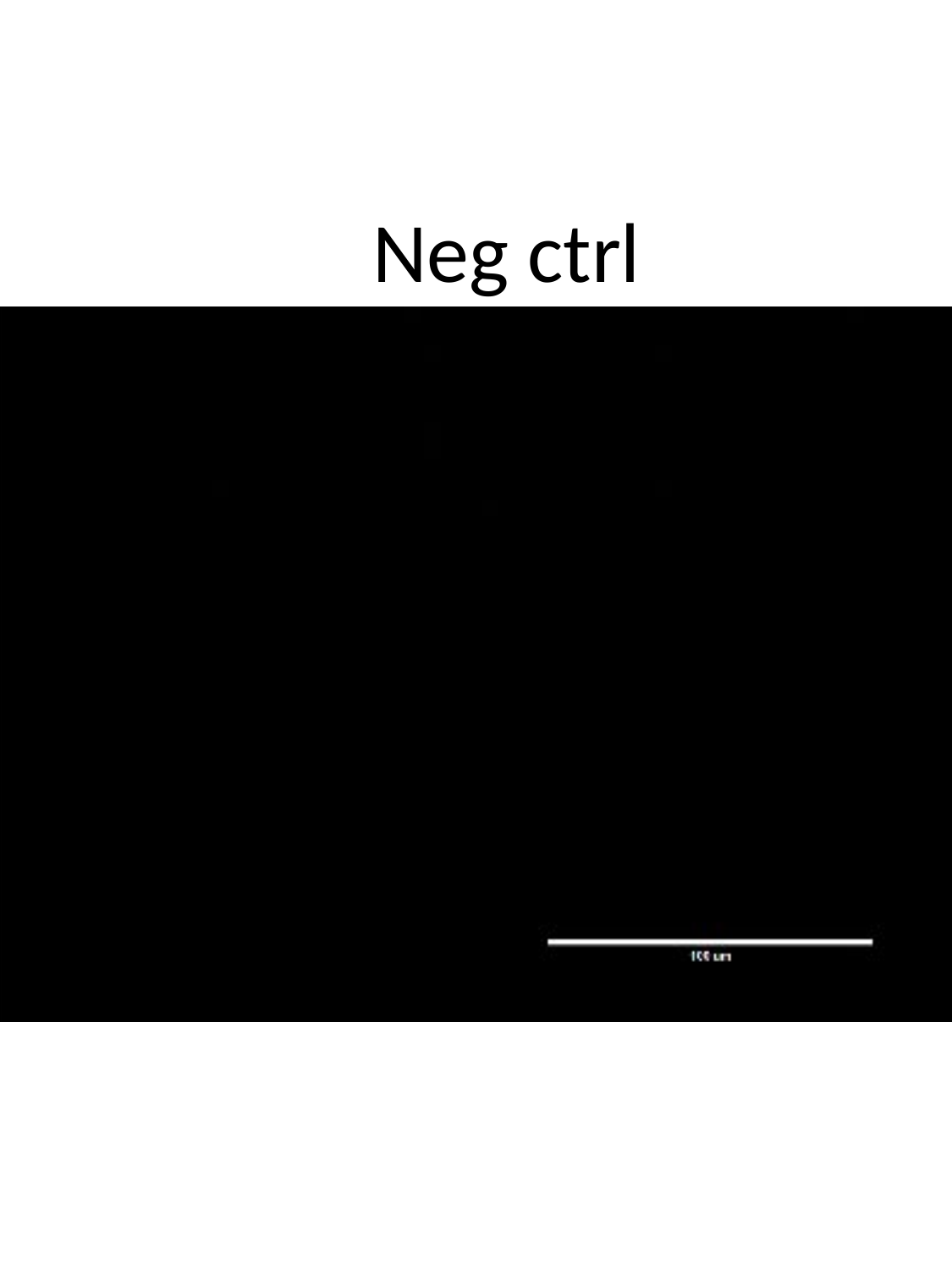

Neg ctrl
