# Supplementary material for: Cardiac differentiation of human pluripotent stem cells using defined extracellular matrix proteins reveals essential role of fibronectin
Source: eLife. 2022 Jun 27;11:e69028. doi: 10.7554/eLife.69028 (PMC9236614; doi:10.7554/eLife.69028)
Supplement: Figure 9—source data 1. [file elife-69028-fig9-data1.zip › Figure 9 WB raw data/Figure 9 WB labeled.pptx]

## Slide 1
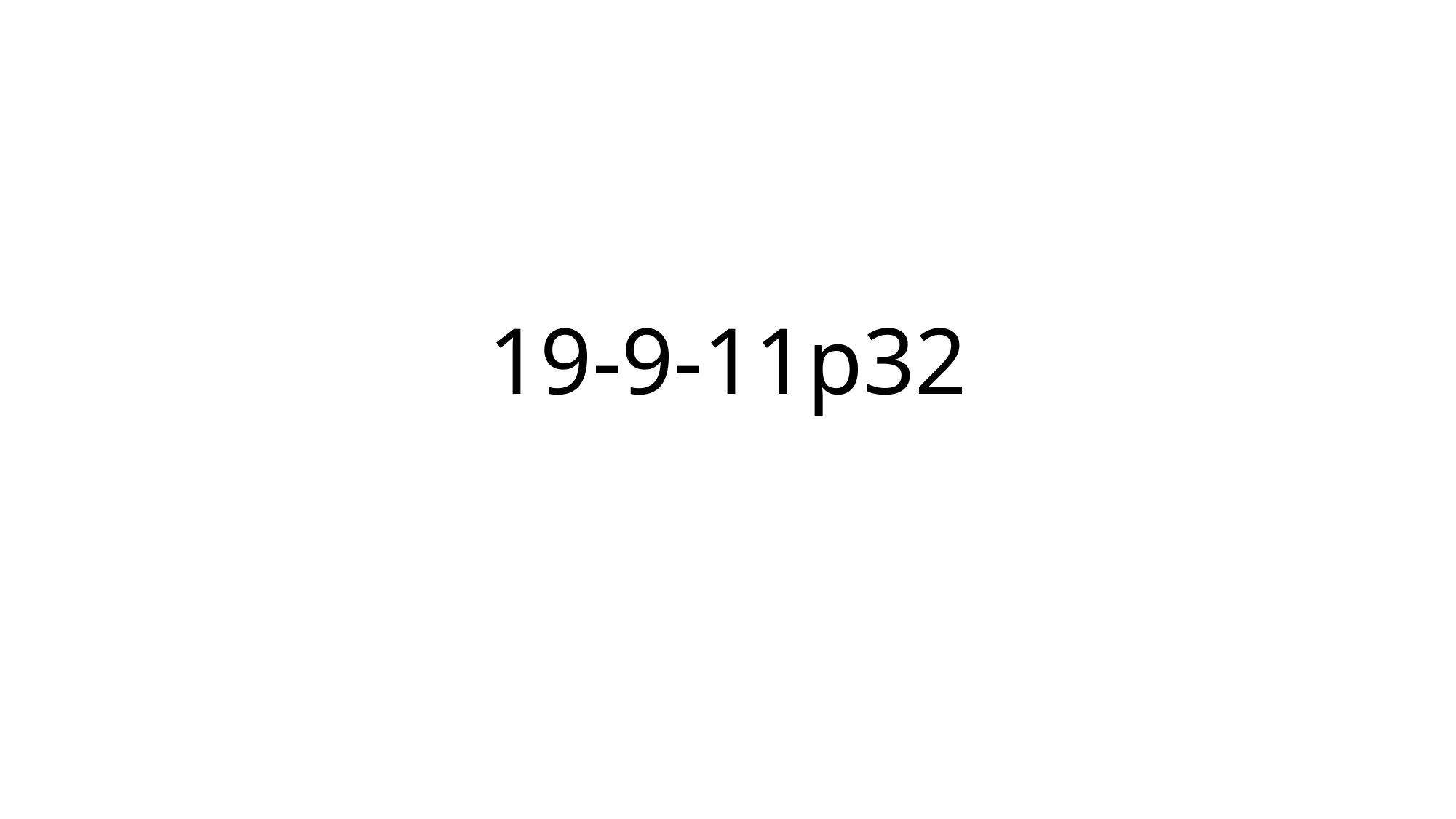

# 19-9-11p32

## Slide 2
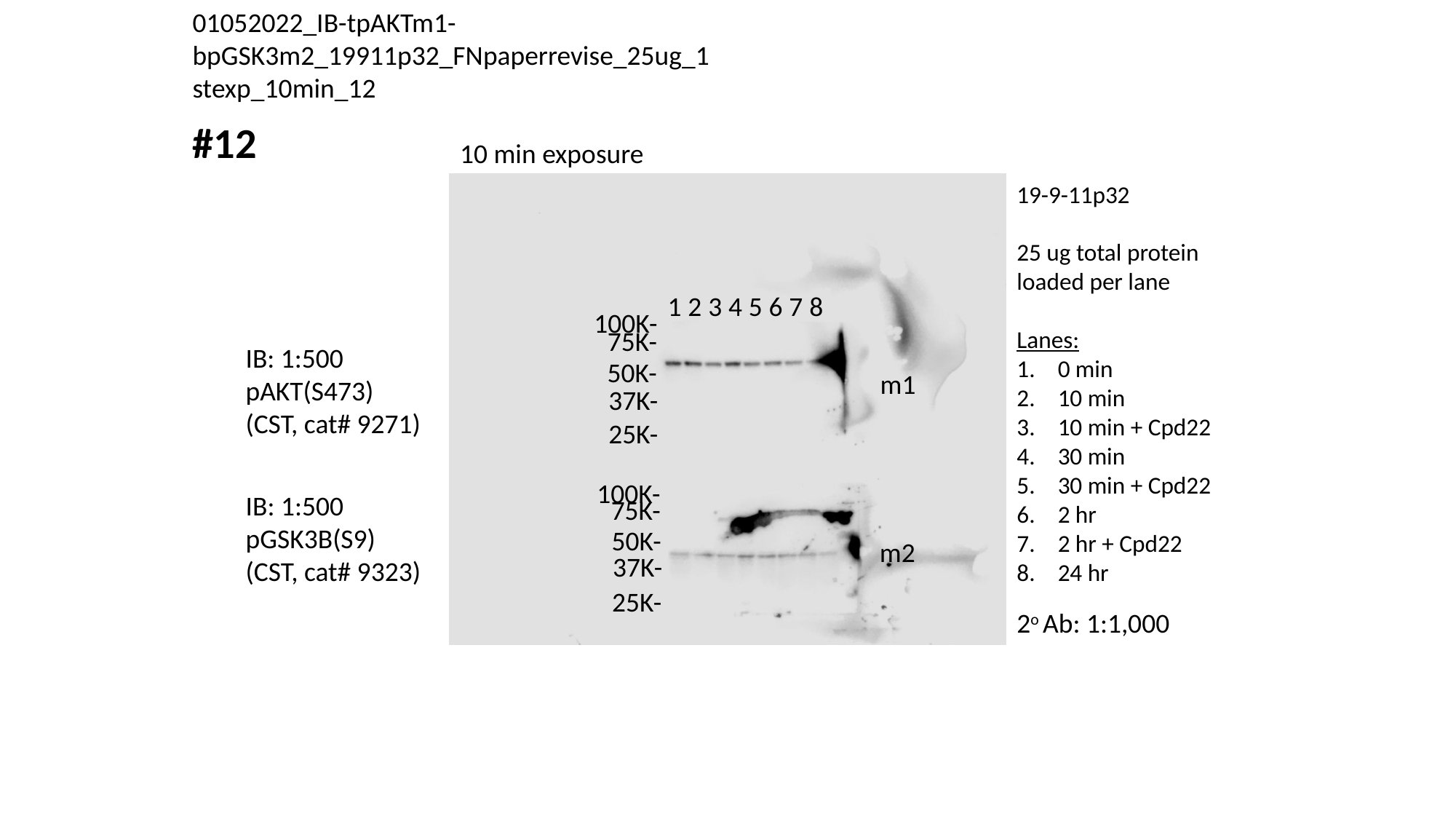

01052022_IB-tpAKTm1-bpGSK3m2_19911p32_FNpaperrevise_25ug_1stexp_10min_12
#12
10 min exposure
19-9-11p32
25 ug total protein loaded per lane
Lanes:
0 min
10 min
10 min + Cpd22
30 min
30 min + Cpd22
2 hr
2 hr + Cpd22
24 hr
1
2
3
4
5
6
7
8
100K-
75K-
IB: 1:500 pAKT(S473)
(CST, cat# 9271)
50K-
m1
37K-
25K-
100K-
IB: 1:500 pGSK3B(S9)
(CST, cat# 9323)
75K-
50K-
m2
37K-
25K-
2o Ab: 1:1,000

## Slide 3
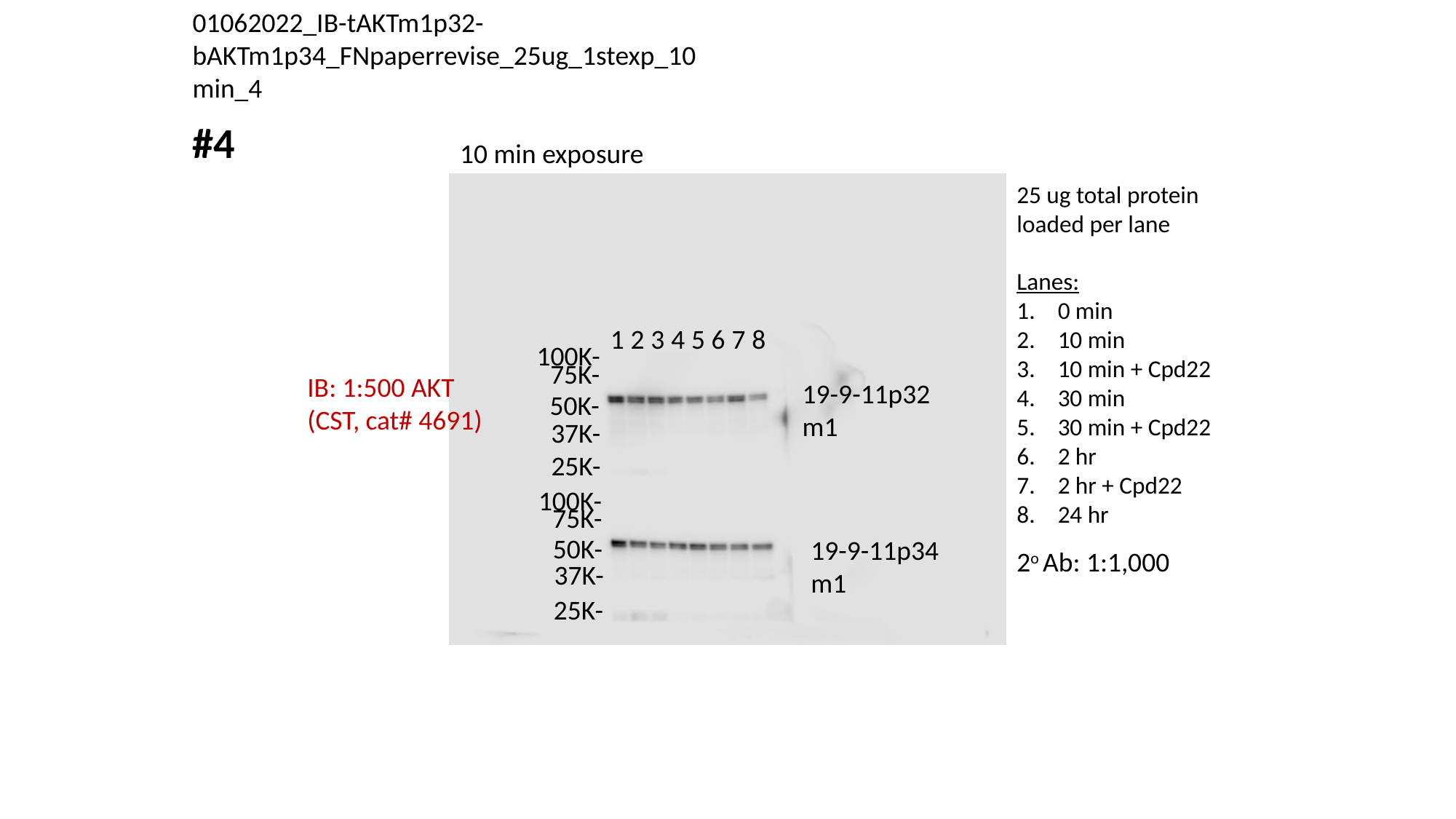

01062022_IB-tAKTm1p32-bAKTm1p34_FNpaperrevise_25ug_1stexp_10min_4
#4
10 min exposure
25 ug total protein loaded per lane
Lanes:
0 min
10 min
10 min + Cpd22
30 min
30 min + Cpd22
2 hr
2 hr + Cpd22
24 hr
1
2
3
4
5
6
7
8
100K-
75K-
IB: 1:500 AKT
(CST, cat# 4691)
19-9-11p32
m1
50K-
37K-
25K-
100K-
75K-
50K-
19-9-11p34
m1
2o Ab: 1:1,000
37K-
25K-

## Slide 4
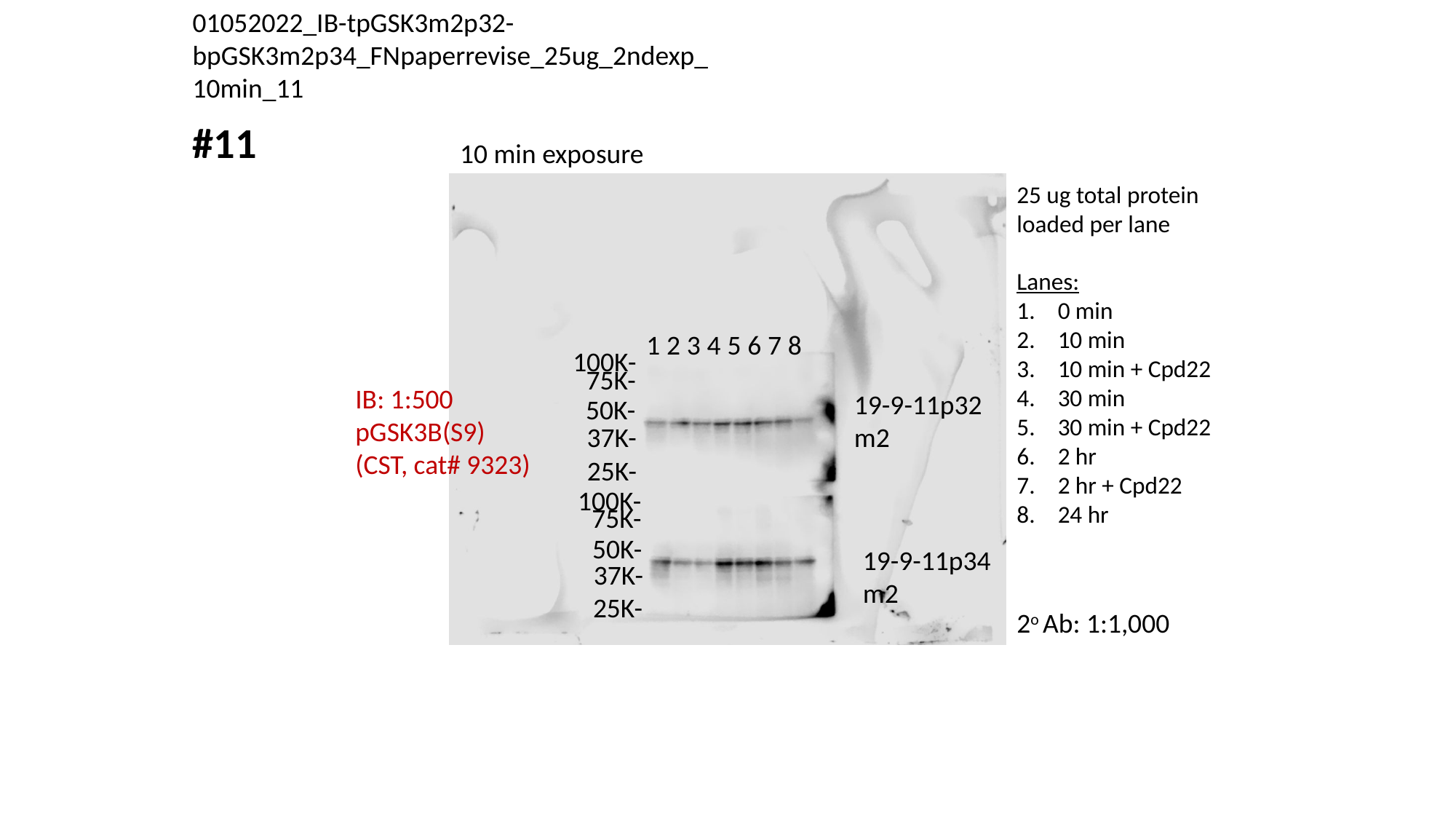

01052022_IB-tpGSK3m2p32-bpGSK3m2p34_FNpaperrevise_25ug_2ndexp_10min_11
#11
10 min exposure
25 ug total protein loaded per lane
Lanes:
0 min
10 min
10 min + Cpd22
30 min
30 min + Cpd22
2 hr
2 hr + Cpd22
24 hr
1
2
3
4
5
6
7
8
100K-
75K-
IB: 1:500 pGSK3B(S9)
(CST, cat# 9323)
19-9-11p32
m2
50K-
37K-
25K-
100K-
75K-
50K-
19-9-11p34
m2
37K-
25K-
2o Ab: 1:1,000

## Slide 5
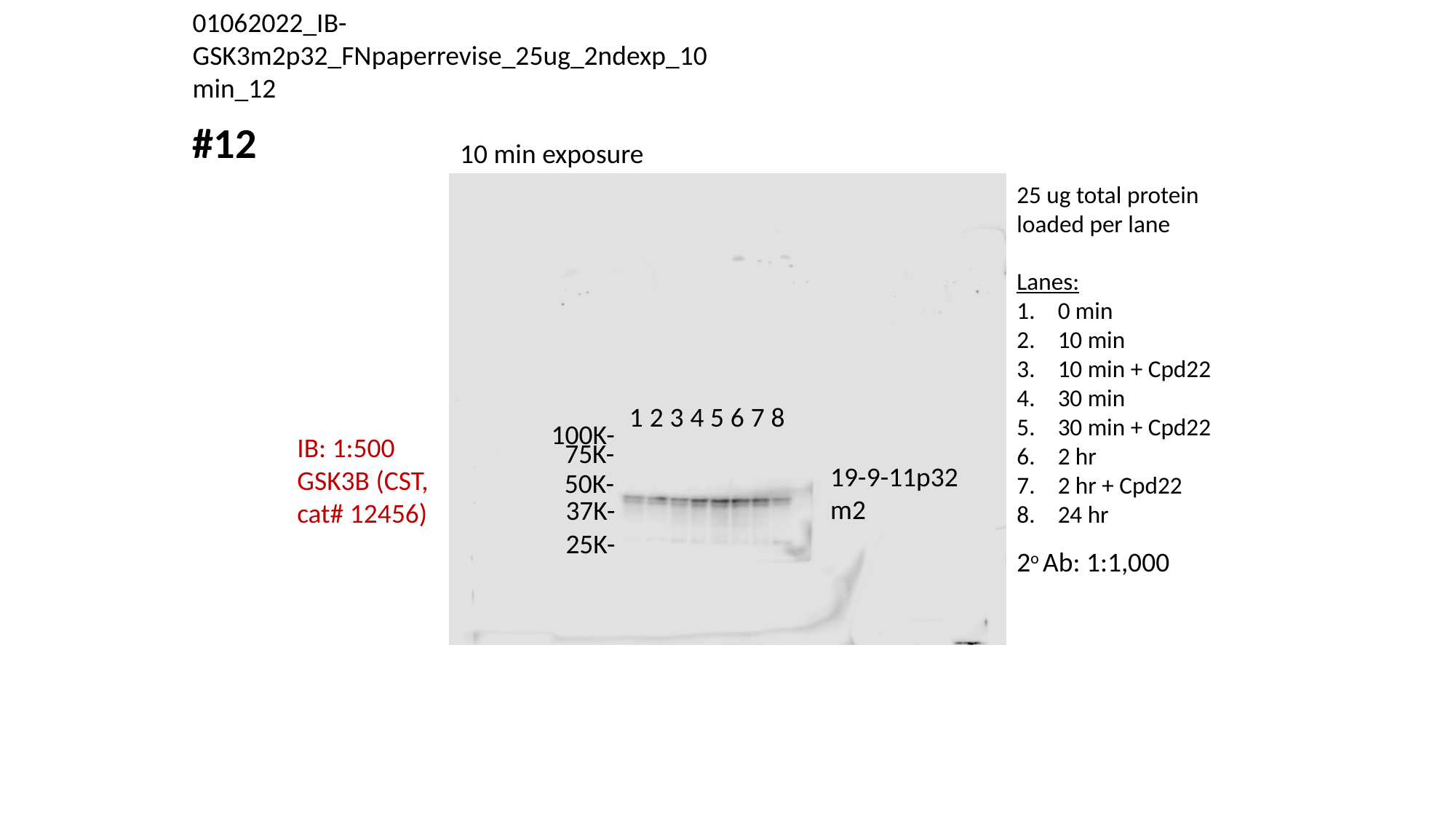

01062022_IB-GSK3m2p32_FNpaperrevise_25ug_2ndexp_10min_12
#12
10 min exposure
25 ug total protein loaded per lane
Lanes:
0 min
10 min
10 min + Cpd22
30 min
30 min + Cpd22
2 hr
2 hr + Cpd22
24 hr
1
2
3
4
5
6
7
8
100K-
IB: 1:500 GSK3B (CST, cat# 12456)
75K-
19-9-11p32
m2
50K-
37K-
25K-
2o Ab: 1:1,000

## Slide 6
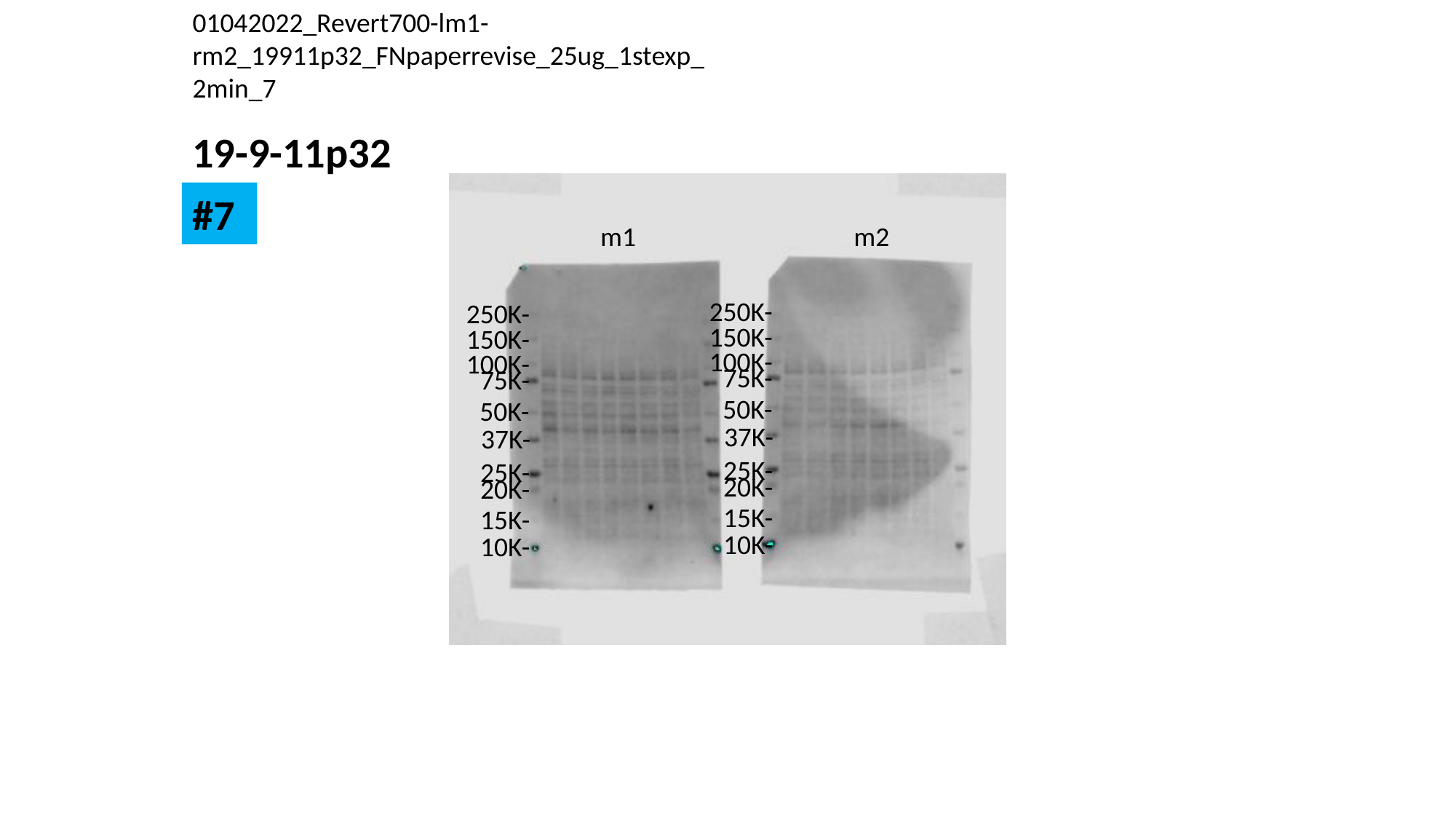

01042022_Revert700-lm1-rm2_19911p32_FNpaperrevise_25ug_1stexp_2min_7
19-9-11p32
#7
m1
m2
250K-
250K-
150K-
150K-
100K-
100K-
75K-
75K-
50K-
50K-
37K-
37K-
25K-
25K-
20K-
20K-
15K-
15K-
10K-
10K-

## Slide 7
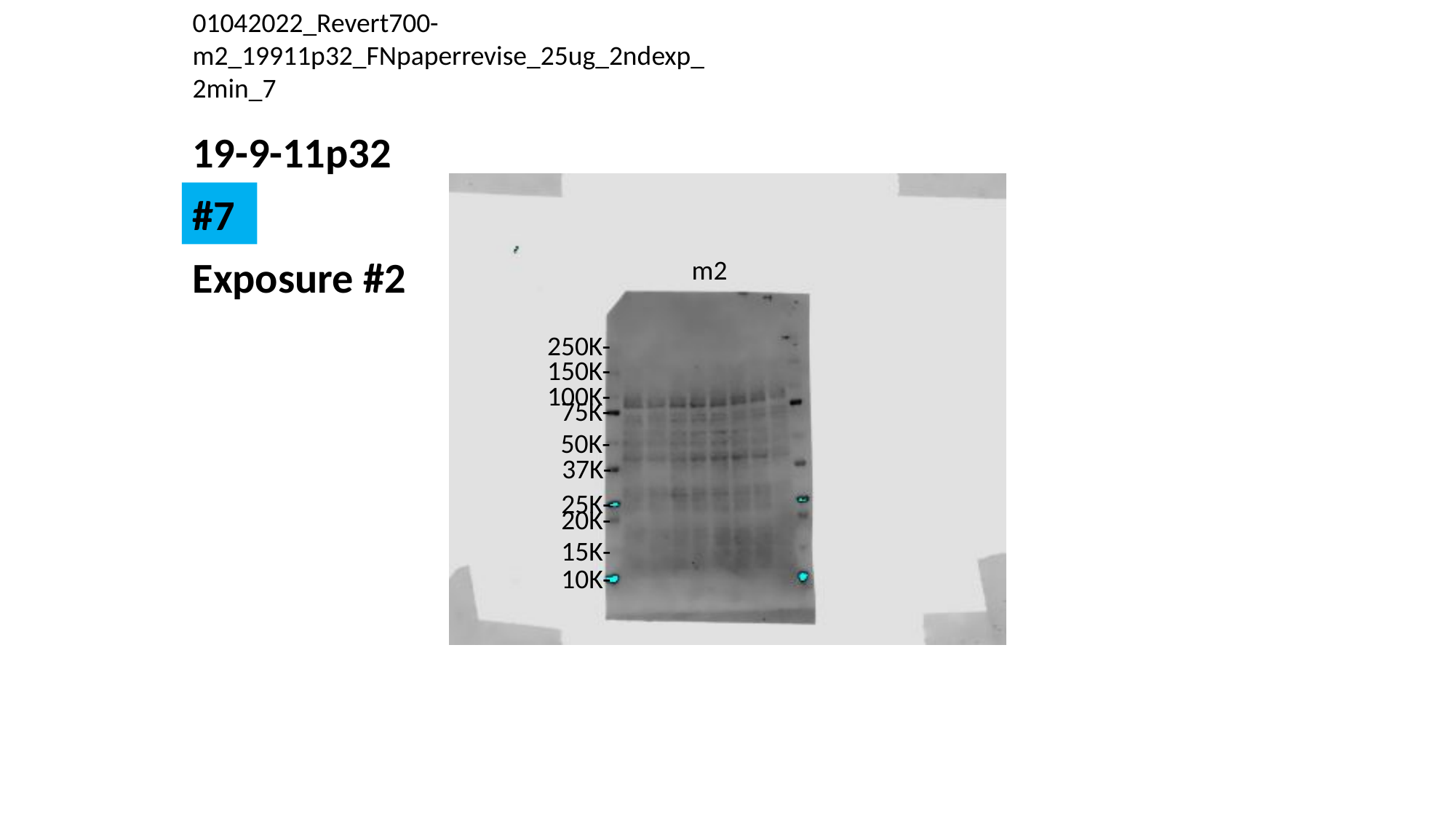

01042022_Revert700-m2_19911p32_FNpaperrevise_25ug_2ndexp_2min_7
19-9-11p32
#7
Exposure #2
m2
250K-
150K-
100K-
75K-
50K-
37K-
25K-
20K-
15K-
10K-

## Slide 8
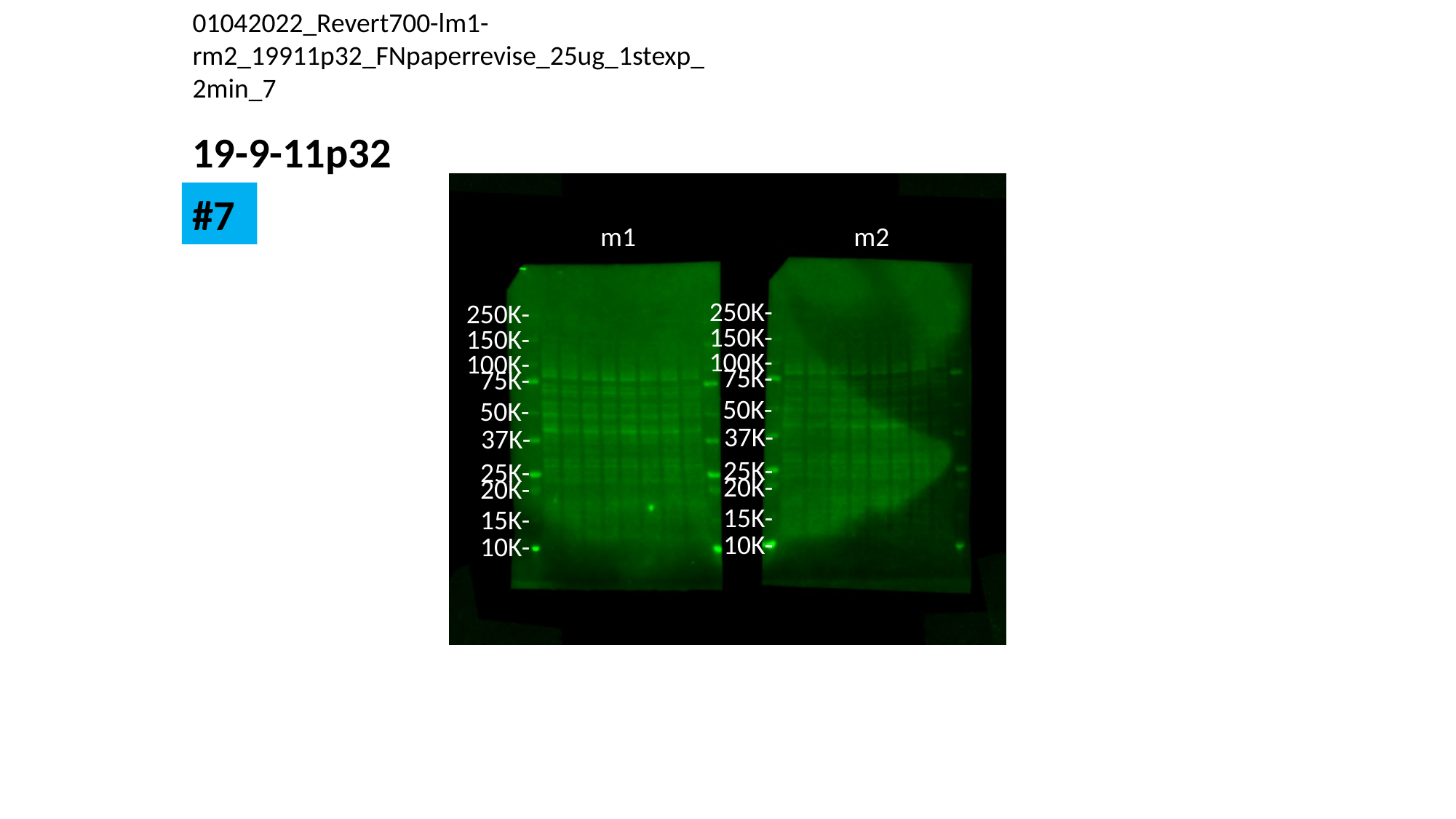

01042022_Revert700-lm1-rm2_19911p32_FNpaperrevise_25ug_1stexp_2min_7
19-9-11p32
#7
m1
m2
250K-
250K-
150K-
150K-
100K-
100K-
75K-
75K-
50K-
50K-
37K-
37K-
25K-
25K-
20K-
20K-
15K-
15K-
10K-
10K-

## Slide 9
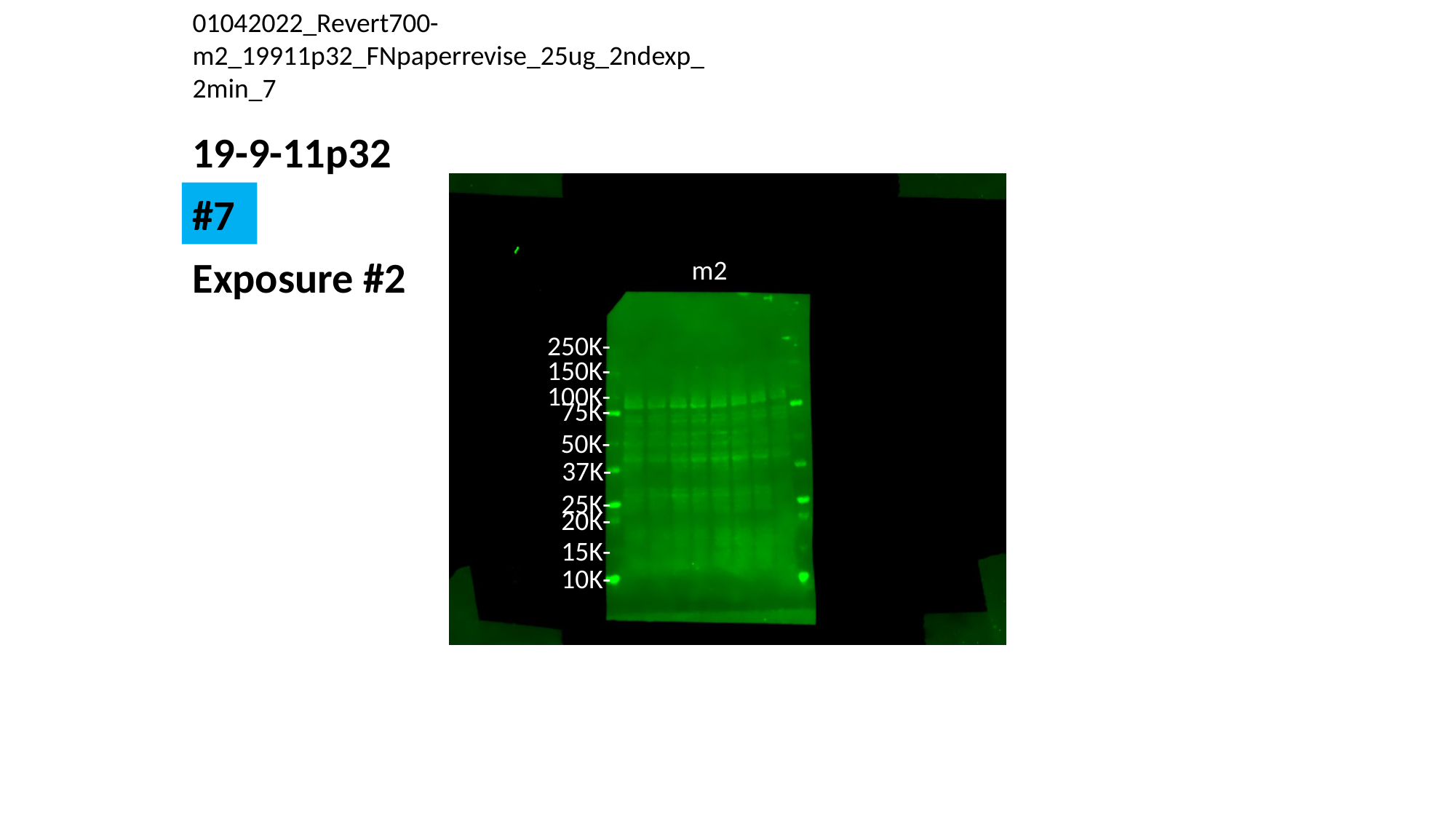

01042022_Revert700-m2_19911p32_FNpaperrevise_25ug_2ndexp_2min_7
19-9-11p32
#7
Exposure #2
m2
250K-
150K-
100K-
75K-
50K-
37K-
25K-
20K-
15K-
10K-

## Slide 10
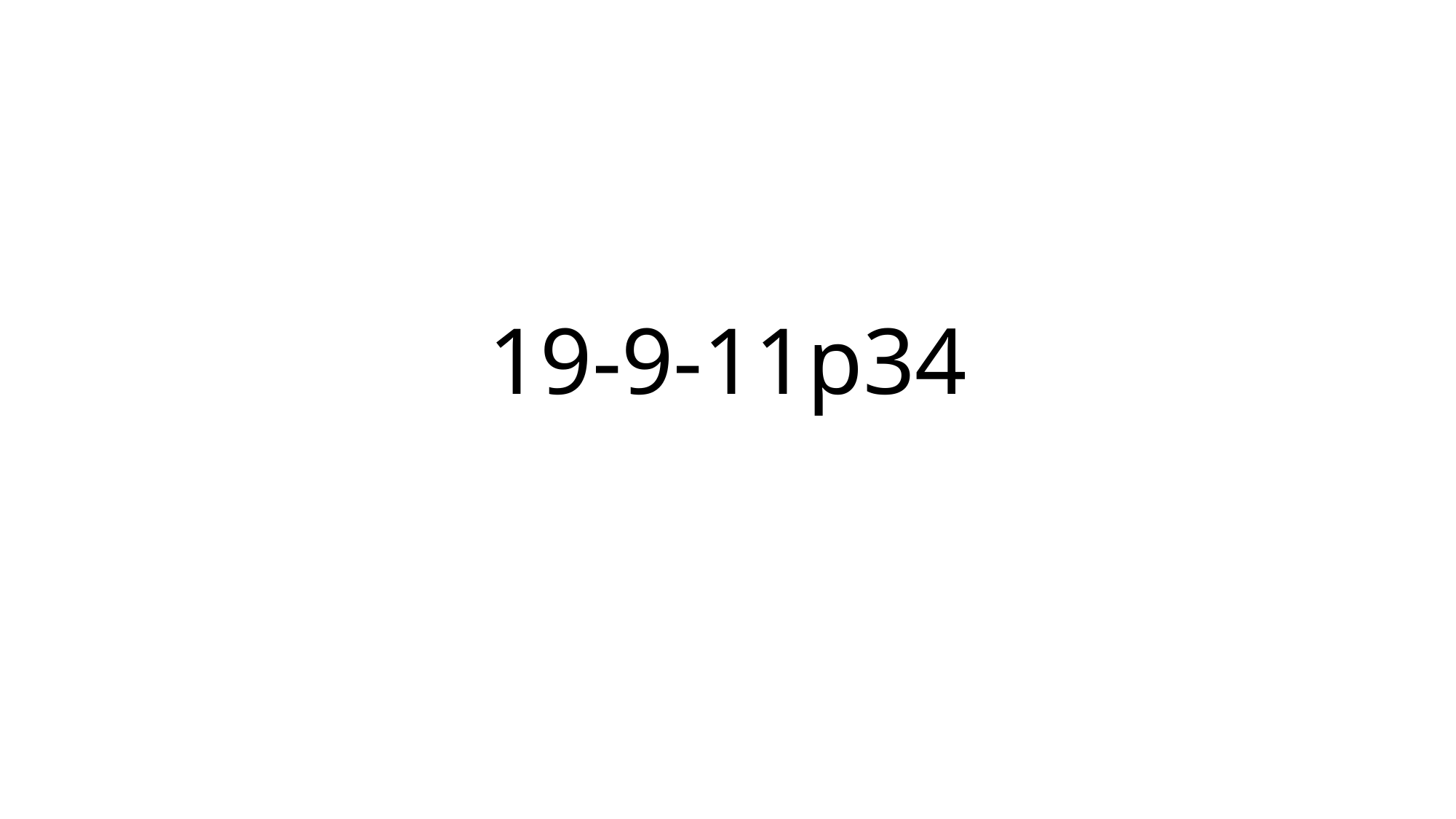

# 19-9-11p34

## Slide 11
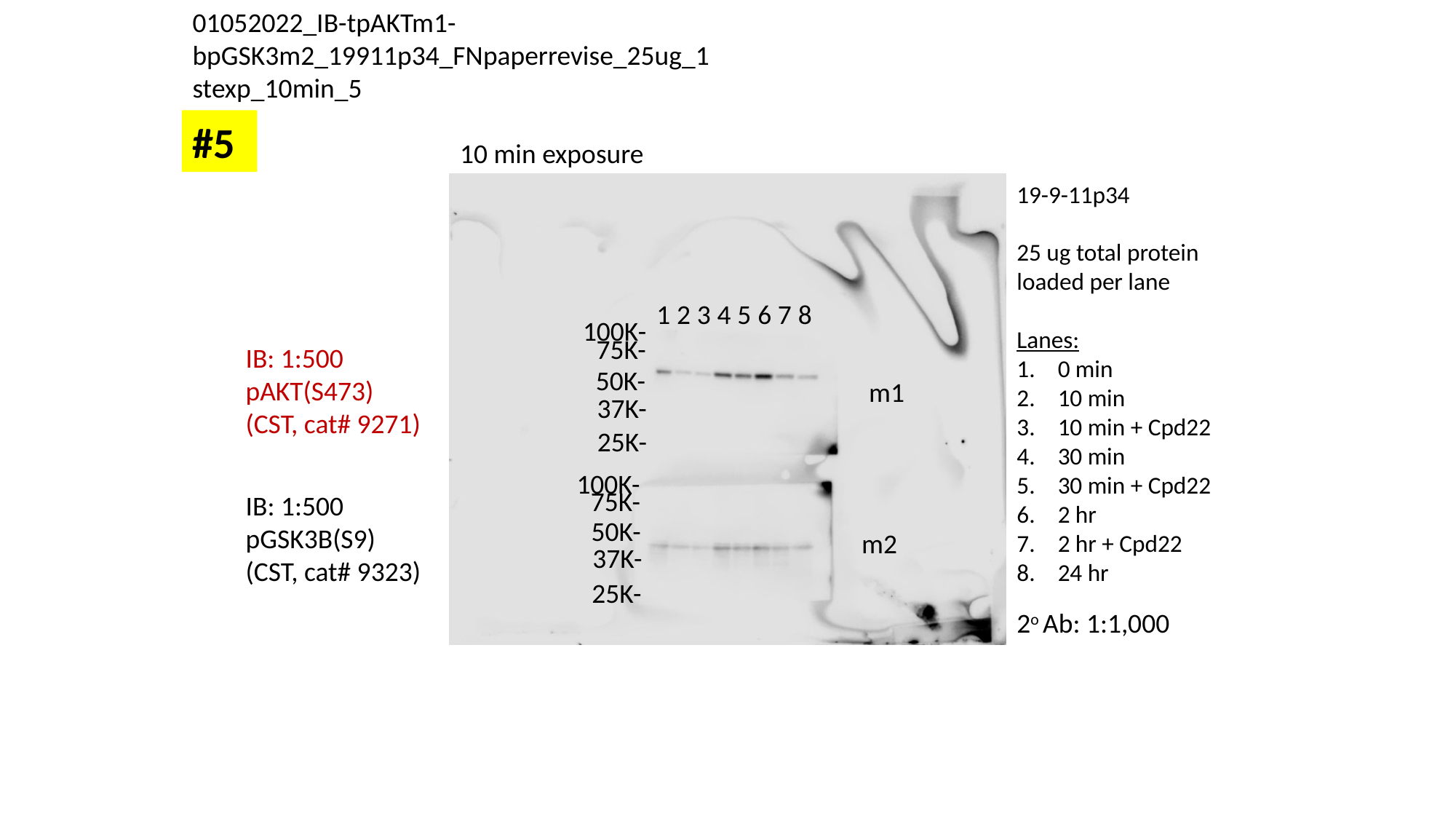

01052022_IB-tpAKTm1-bpGSK3m2_19911p34_FNpaperrevise_25ug_1stexp_10min_5
#5
10 min exposure
19-9-11p34
25 ug total protein loaded per lane
Lanes:
0 min
10 min
10 min + Cpd22
30 min
30 min + Cpd22
2 hr
2 hr + Cpd22
24 hr
1
2
3
4
5
6
7
8
100K-
75K-
IB: 1:500 pAKT(S473)
(CST, cat# 9271)
50K-
m1
37K-
25K-
100K-
75K-
IB: 1:500 pGSK3B(S9)
(CST, cat# 9323)
50K-
m2
37K-
25K-
2o Ab: 1:1,000

## Slide 12
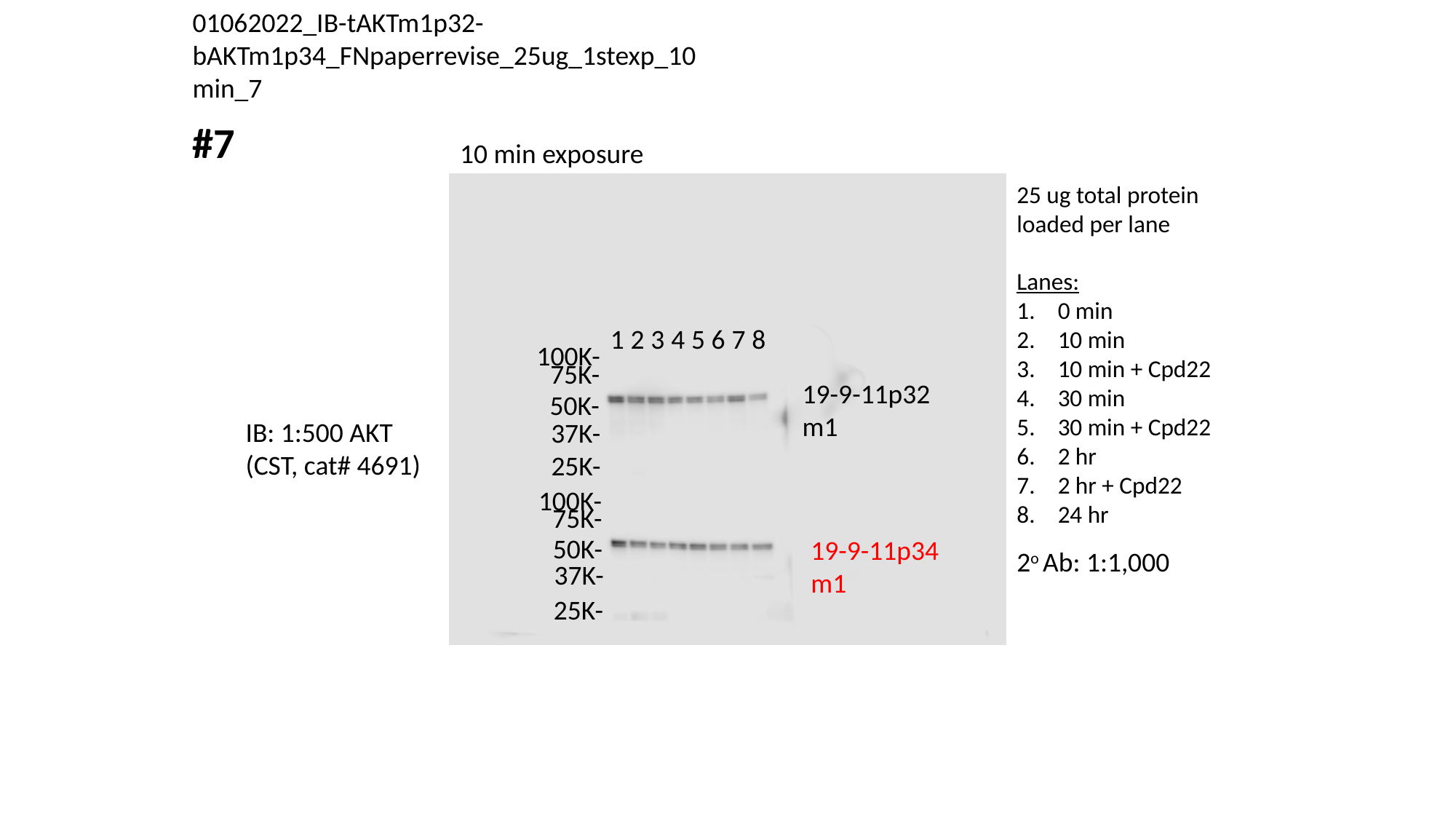

01062022_IB-tAKTm1p32-bAKTm1p34_FNpaperrevise_25ug_1stexp_10min_7
#7
10 min exposure
25 ug total protein loaded per lane
Lanes:
0 min
10 min
10 min + Cpd22
30 min
30 min + Cpd22
2 hr
2 hr + Cpd22
24 hr
1
2
3
4
5
6
7
8
100K-
75K-
19-9-11p32
m1
50K-
IB: 1:500 AKT
(CST, cat# 4691)
37K-
25K-
100K-
75K-
50K-
19-9-11p34
m1
2o Ab: 1:1,000
37K-
25K-

## Slide 13
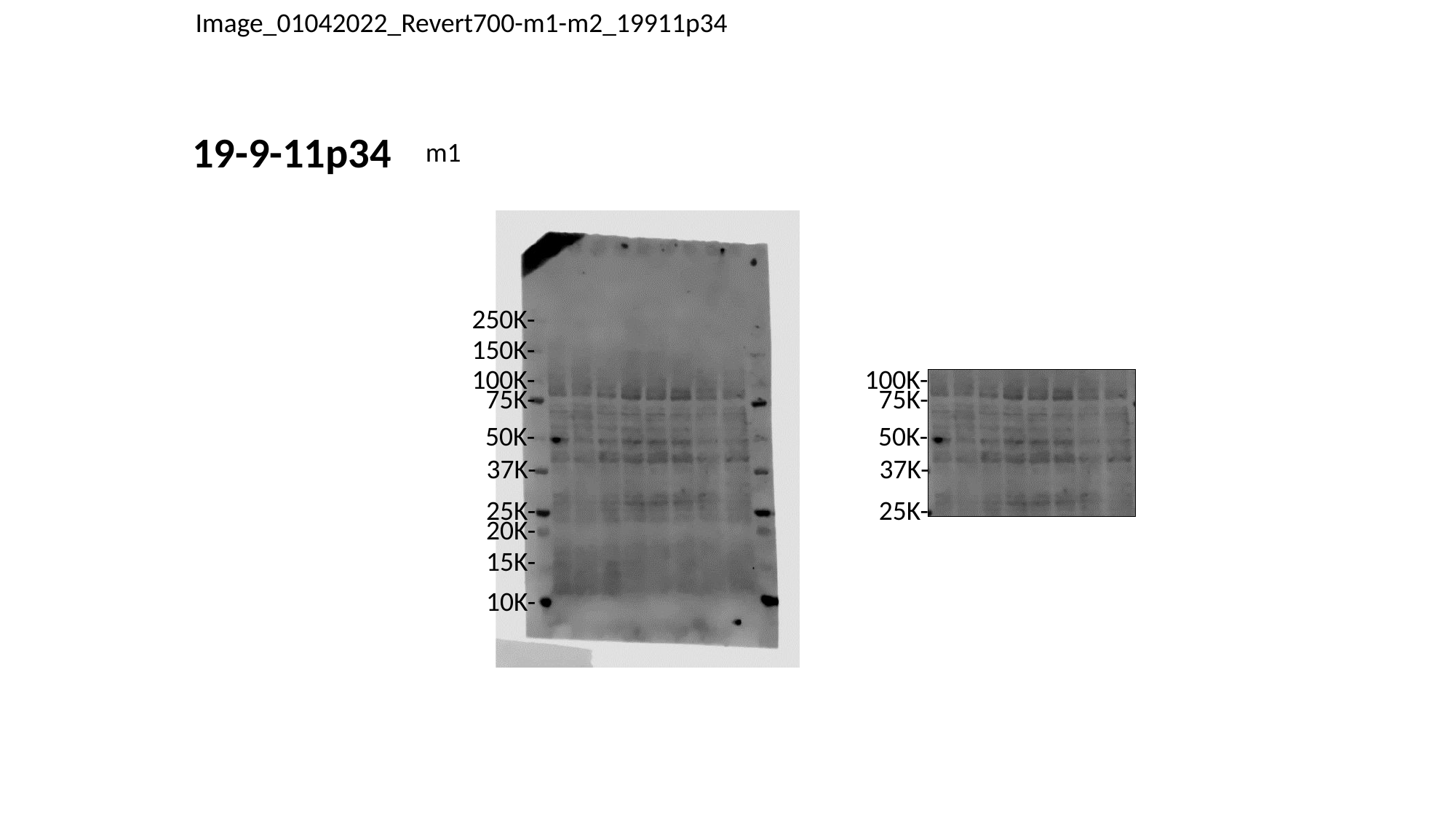

Image_01042022_Revert700-m1-m2_19911p34
19-9-11p34
m1
250K-
150K-
100K-
100K-
75K-
75K-
50K-
50K-
37K-
37K-
25K-
25K-
20K-
15K-
10K-

## Slide 14
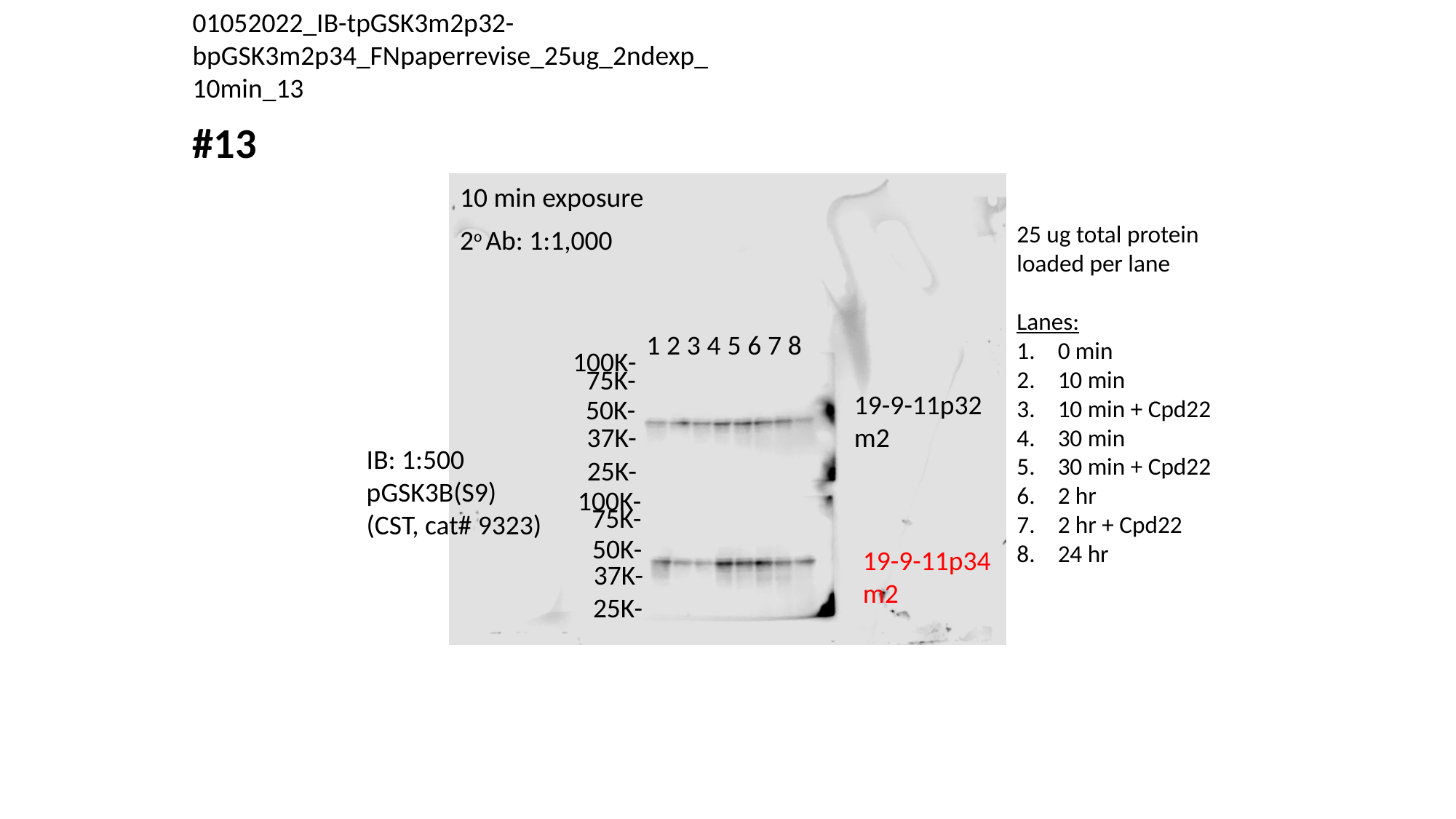

01052022_IB-tpGSK3m2p32-bpGSK3m2p34_FNpaperrevise_25ug_2ndexp_10min_13
#13
10 min exposure
25 ug total protein loaded per lane
Lanes:
0 min
10 min
10 min + Cpd22
30 min
30 min + Cpd22
2 hr
2 hr + Cpd22
24 hr
2o Ab: 1:1,000
1
2
3
4
5
6
7
8
100K-
75K-
19-9-11p32
m2
50K-
37K-
IB: 1:500 pGSK3B(S9)
(CST, cat# 9323)
25K-
100K-
75K-
50K-
19-9-11p34
m2
37K-
25K-

## Slide 15
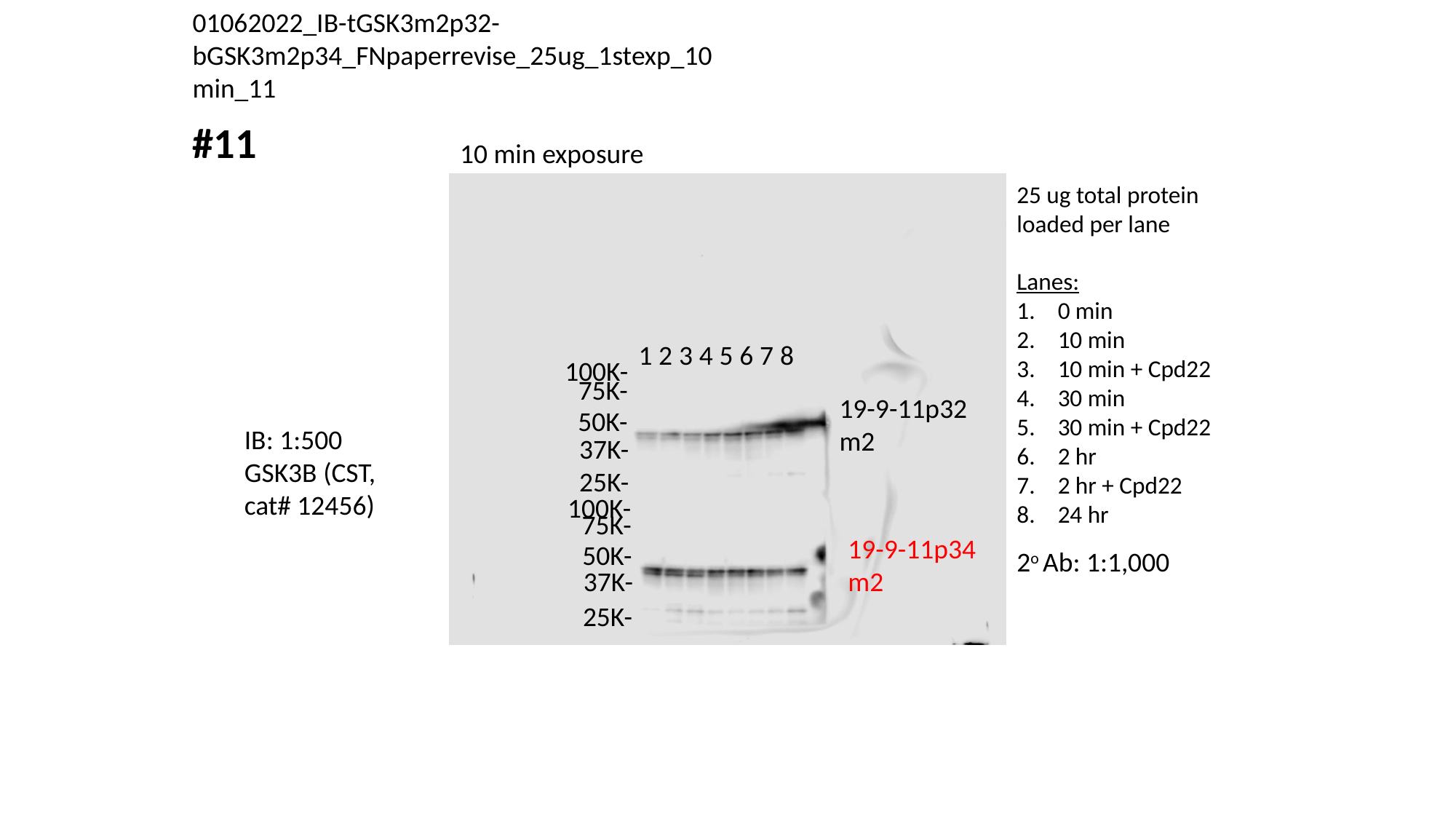

01062022_IB-tGSK3m2p32-bGSK3m2p34_FNpaperrevise_25ug_1stexp_10min_11
#11
10 min exposure
25 ug total protein loaded per lane
Lanes:
0 min
10 min
10 min + Cpd22
30 min
30 min + Cpd22
2 hr
2 hr + Cpd22
24 hr
1
2
3
4
5
6
7
8
100K-
75K-
19-9-11p32
m2
50K-
IB: 1:500 GSK3B (CST, cat# 12456)
37K-
25K-
100K-
75K-
19-9-11p34
m2
50K-
2o Ab: 1:1,000
37K-
25K-

## Slide 16
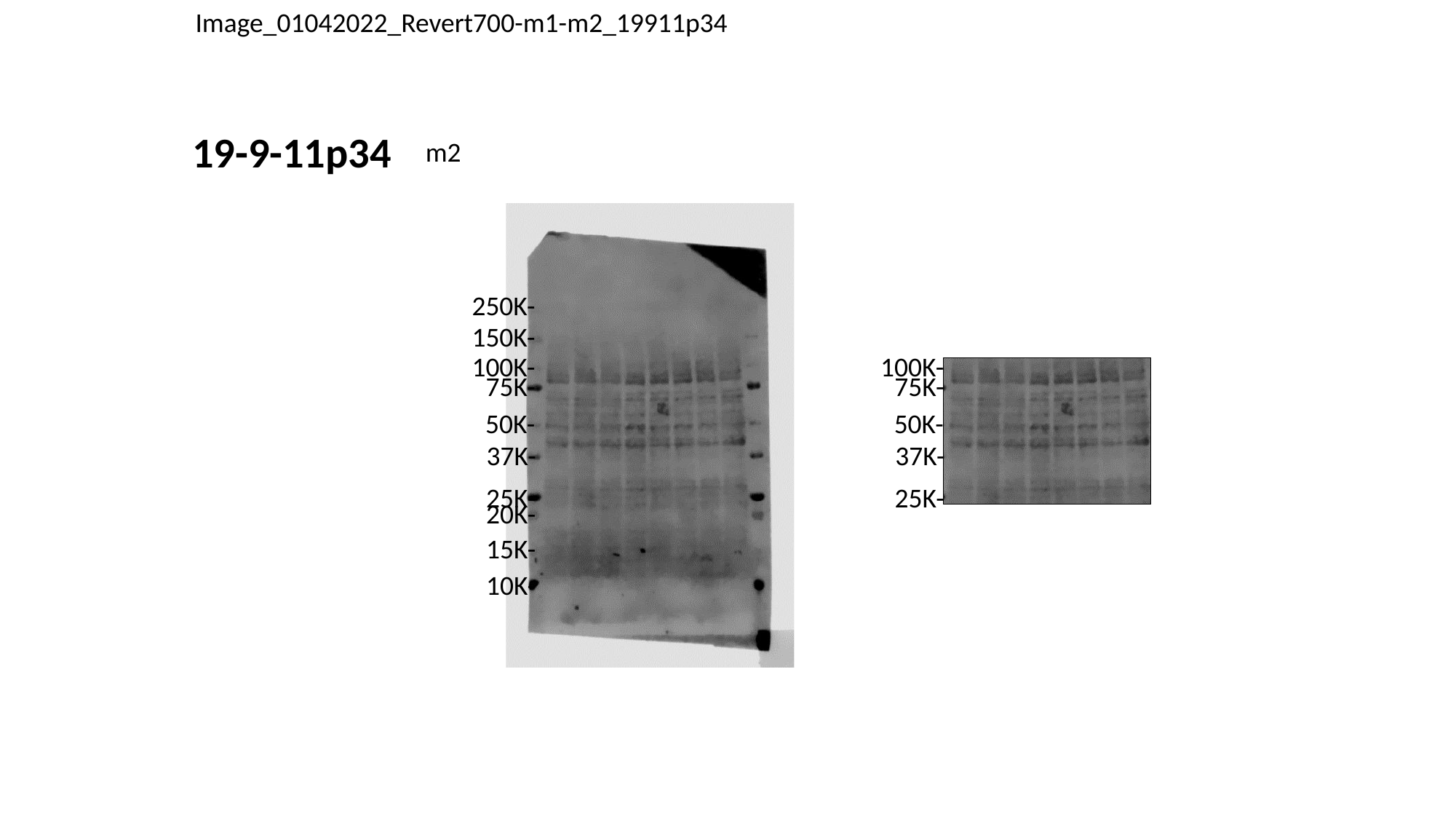

Image_01042022_Revert700-m1-m2_19911p34
19-9-11p34
m2
250K-
150K-
100K-
100K-
75K-
75K-
50K-
50K-
37K-
37K-
25K-
25K-
20K-
15K-
10K-

## Slide 17
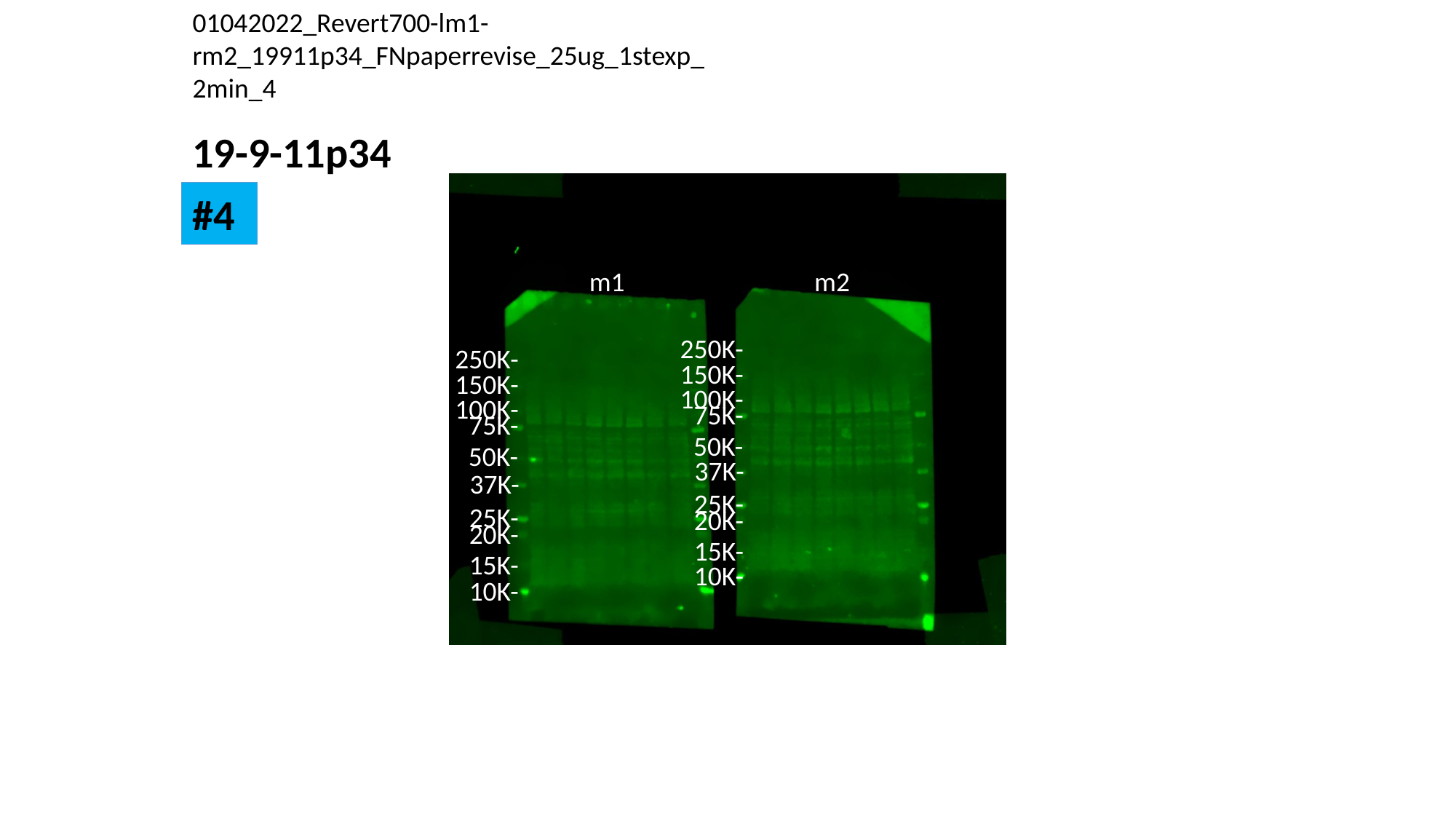

01042022_Revert700-lm1-rm2_19911p34_FNpaperrevise_25ug_1stexp_2min_4
19-9-11p34
#4
m1
m2
250K-
250K-
150K-
150K-
100K-
100K-
75K-
75K-
50K-
50K-
37K-
37K-
25K-
25K-
20K-
20K-
15K-
15K-
10K-
10K-

## Slide 18
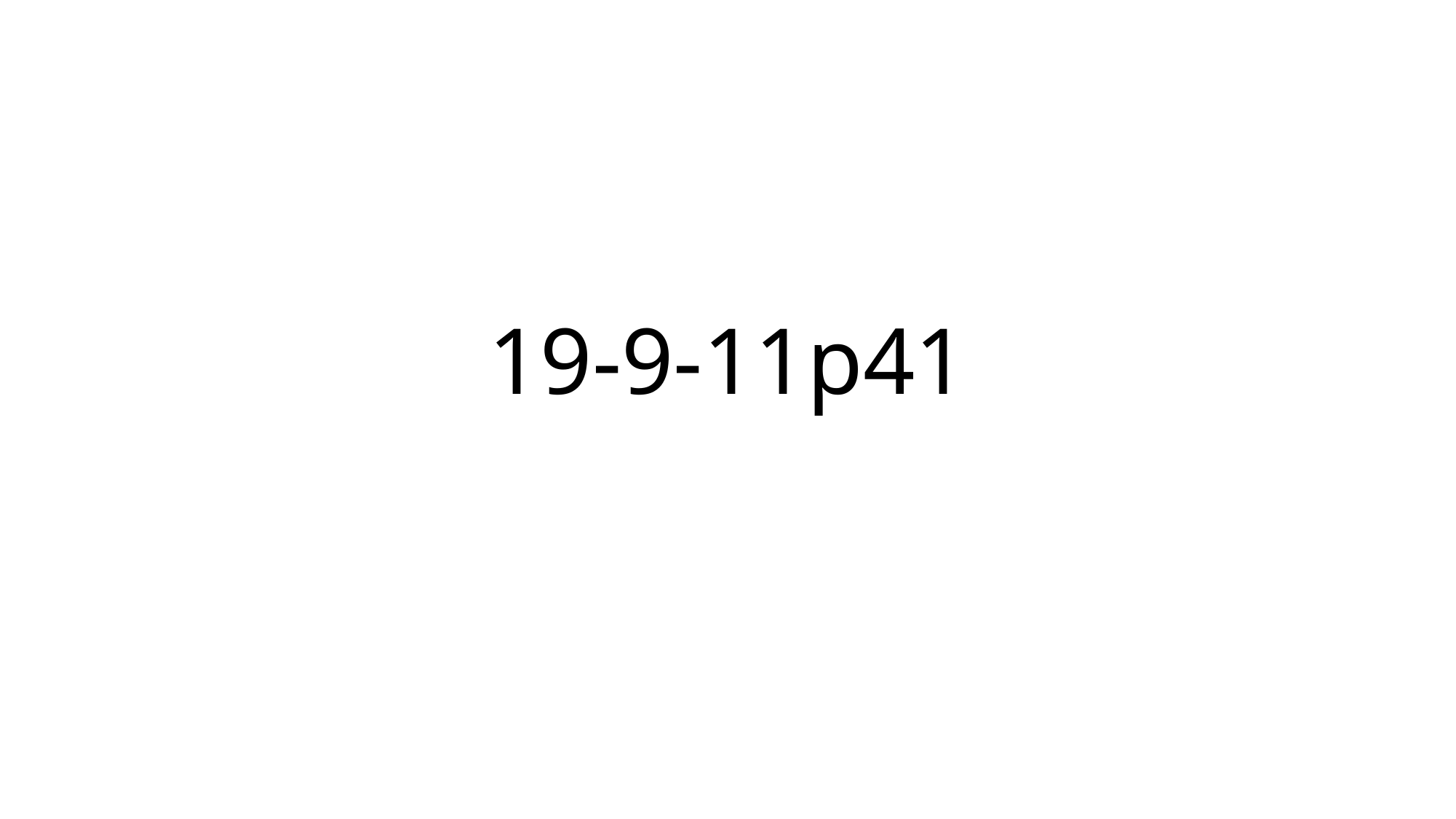

# 19-9-11p41

## Slide 19
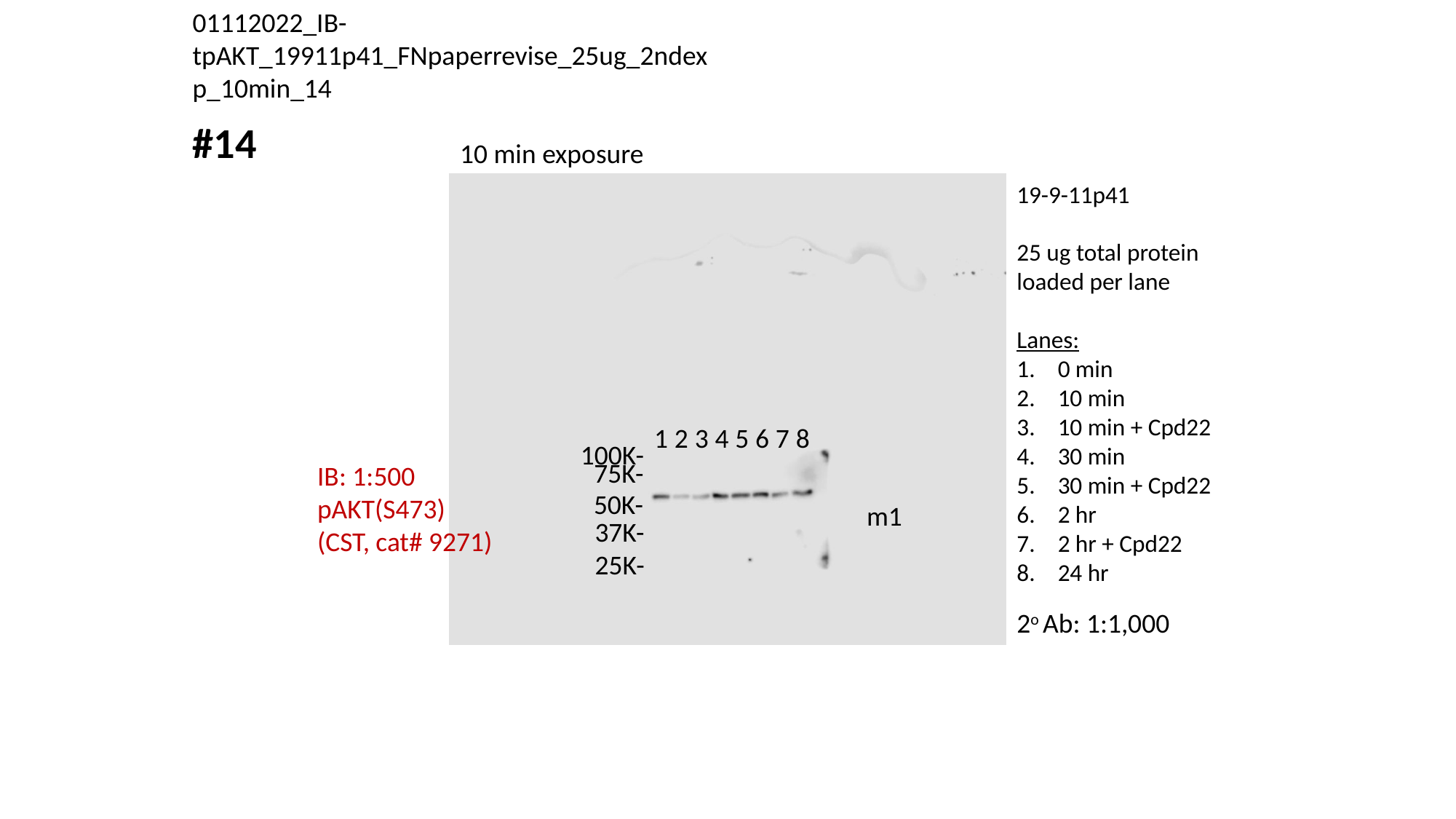

01112022_IB-tpAKT_19911p41_FNpaperrevise_25ug_2ndexp_10min_14
#14
10 min exposure
19-9-11p41
25 ug total protein loaded per lane
Lanes:
0 min
10 min
10 min + Cpd22
30 min
30 min + Cpd22
2 hr
2 hr + Cpd22
24 hr
1
2
3
4
5
6
7
8
100K-
75K-
IB: 1:500 pAKT(S473)
(CST, cat# 9271)
50K-
m1
37K-
25K-
2o Ab: 1:1,000

## Slide 20
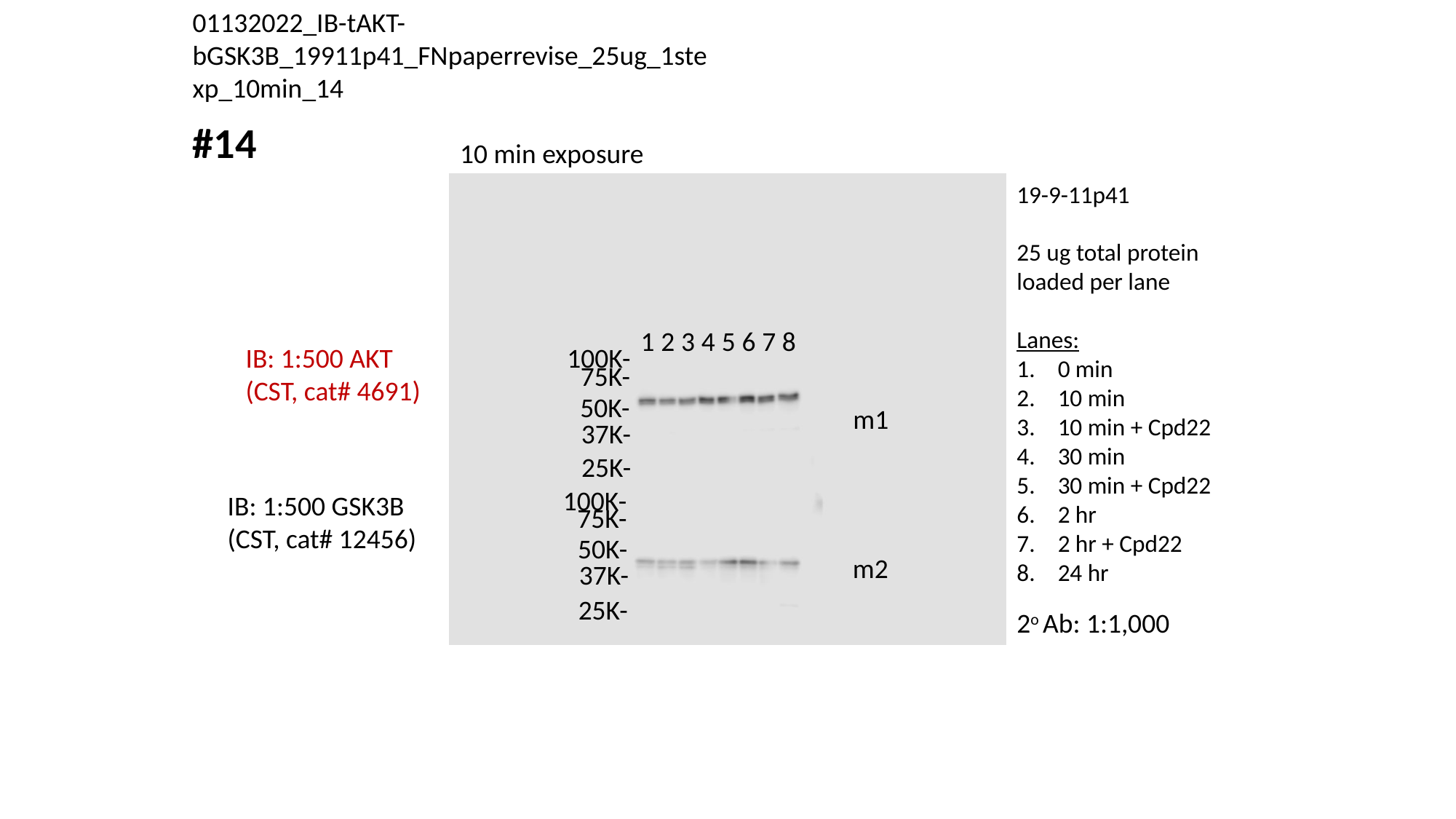

01132022_IB-tAKT-bGSK3B_19911p41_FNpaperrevise_25ug_1stexp_10min_14
#14
10 min exposure
19-9-11p41
25 ug total protein loaded per lane
Lanes:
0 min
10 min
10 min + Cpd22
30 min
30 min + Cpd22
2 hr
2 hr + Cpd22
24 hr
1
2
3
4
5
6
7
8
100K-
IB: 1:500 AKT (CST, cat# 4691)
75K-
50K-
m1
37K-
25K-
100K-
IB: 1:500 GSK3B (CST, cat# 12456)
75K-
50K-
m2
37K-
25K-
2o Ab: 1:1,000

## Slide 21
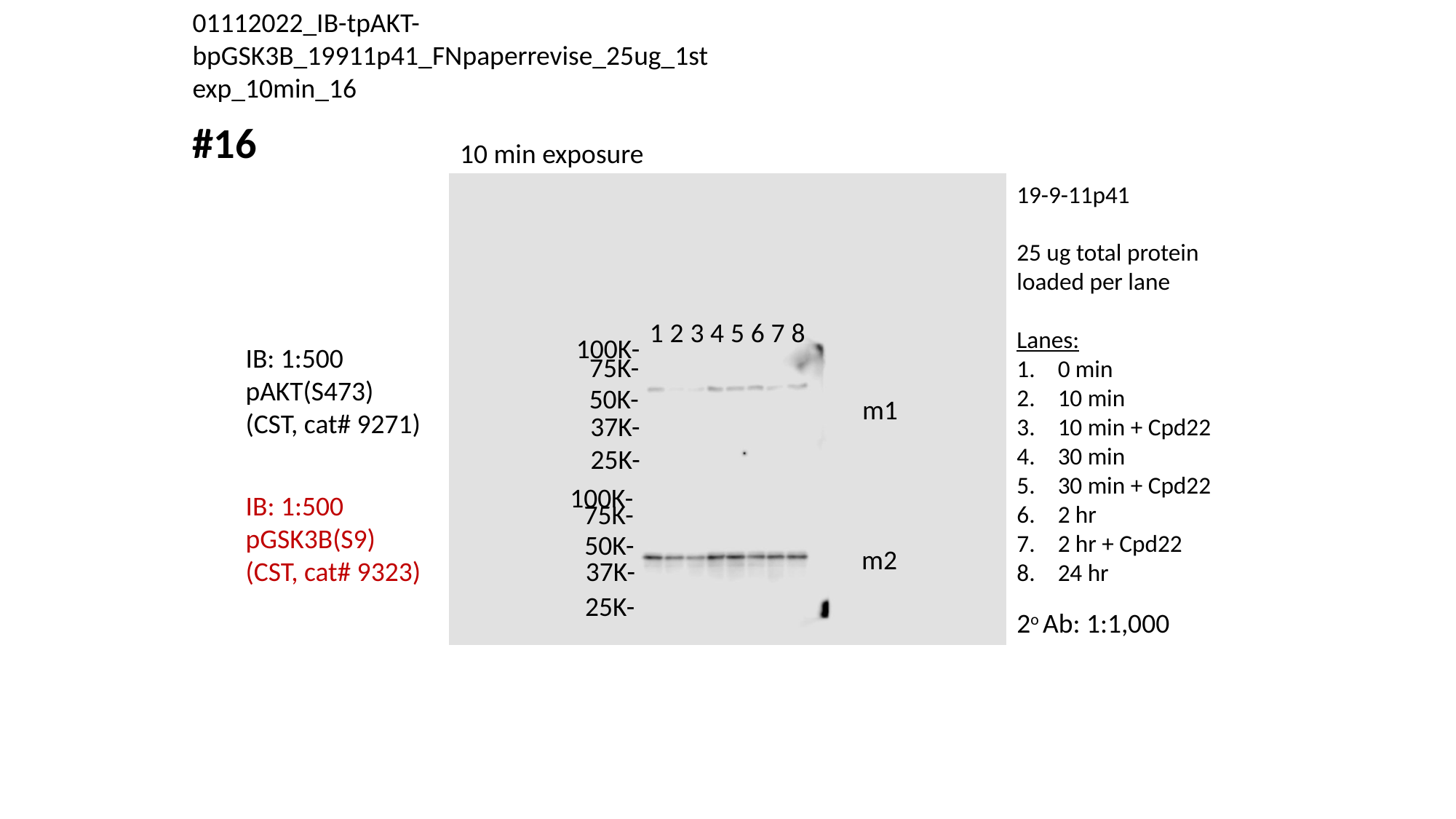

01112022_IB-tpAKT-bpGSK3B_19911p41_FNpaperrevise_25ug_1stexp_10min_16
#16
10 min exposure
19-9-11p41
25 ug total protein loaded per lane
Lanes:
0 min
10 min
10 min + Cpd22
30 min
30 min + Cpd22
2 hr
2 hr + Cpd22
24 hr
1
2
3
4
5
6
7
8
100K-
IB: 1:500 pAKT(S473)
(CST, cat# 9271)
75K-
50K-
m1
37K-
25K-
100K-
IB: 1:500 pGSK3B(S9)
(CST, cat# 9323)
75K-
50K-
m2
37K-
25K-
2o Ab: 1:1,000

## Slide 22
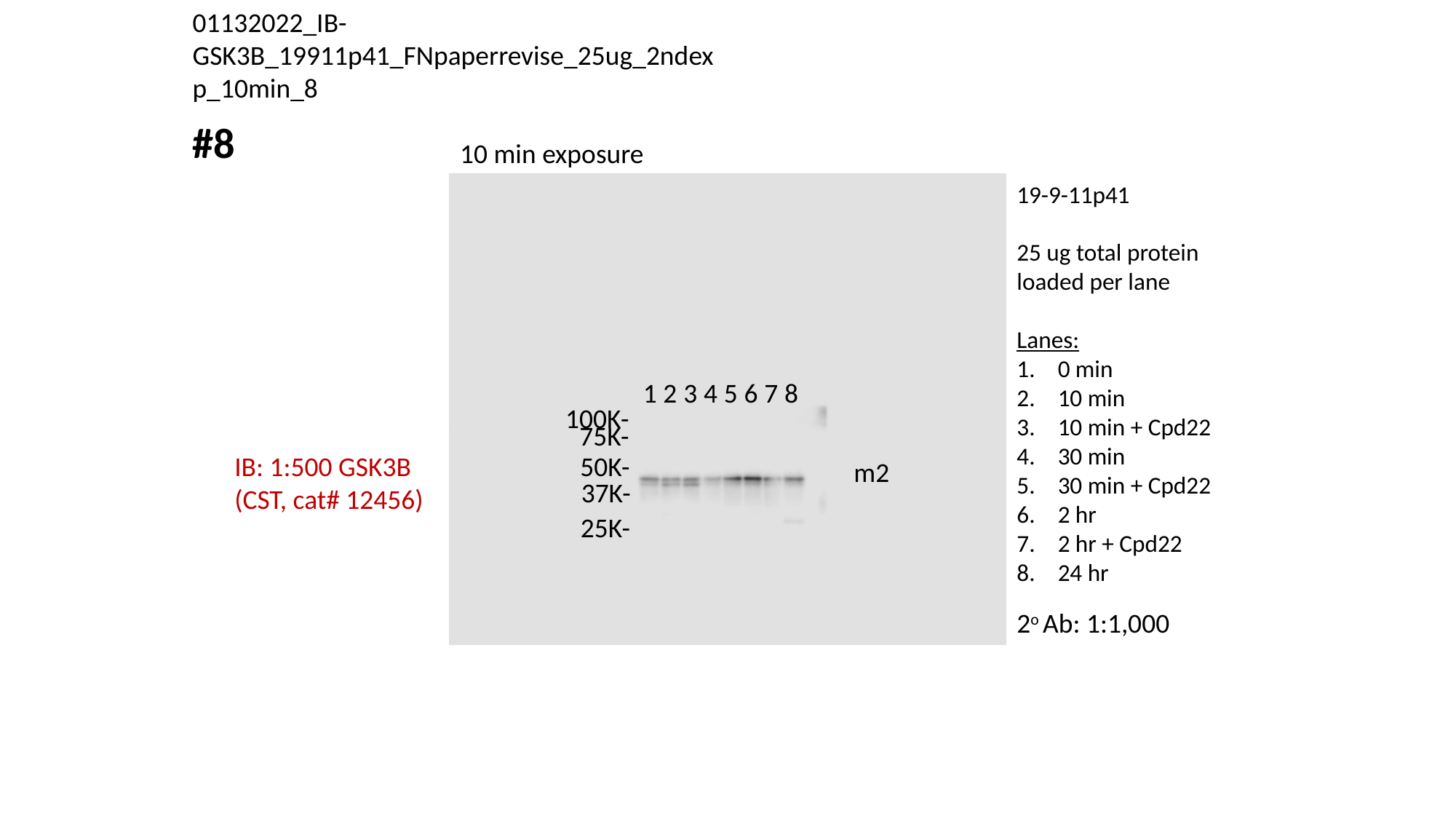

01132022_IB-GSK3B_19911p41_FNpaperrevise_25ug_2ndexp_10min_8
#8
10 min exposure
19-9-11p41
25 ug total protein loaded per lane
Lanes:
0 min
10 min
10 min + Cpd22
30 min
30 min + Cpd22
2 hr
2 hr + Cpd22
24 hr
1
2
3
4
5
6
7
8
100K-
75K-
50K-
IB: 1:500 GSK3B (CST, cat# 12456)
m2
37K-
25K-
2o Ab: 1:1,000

## Slide 23
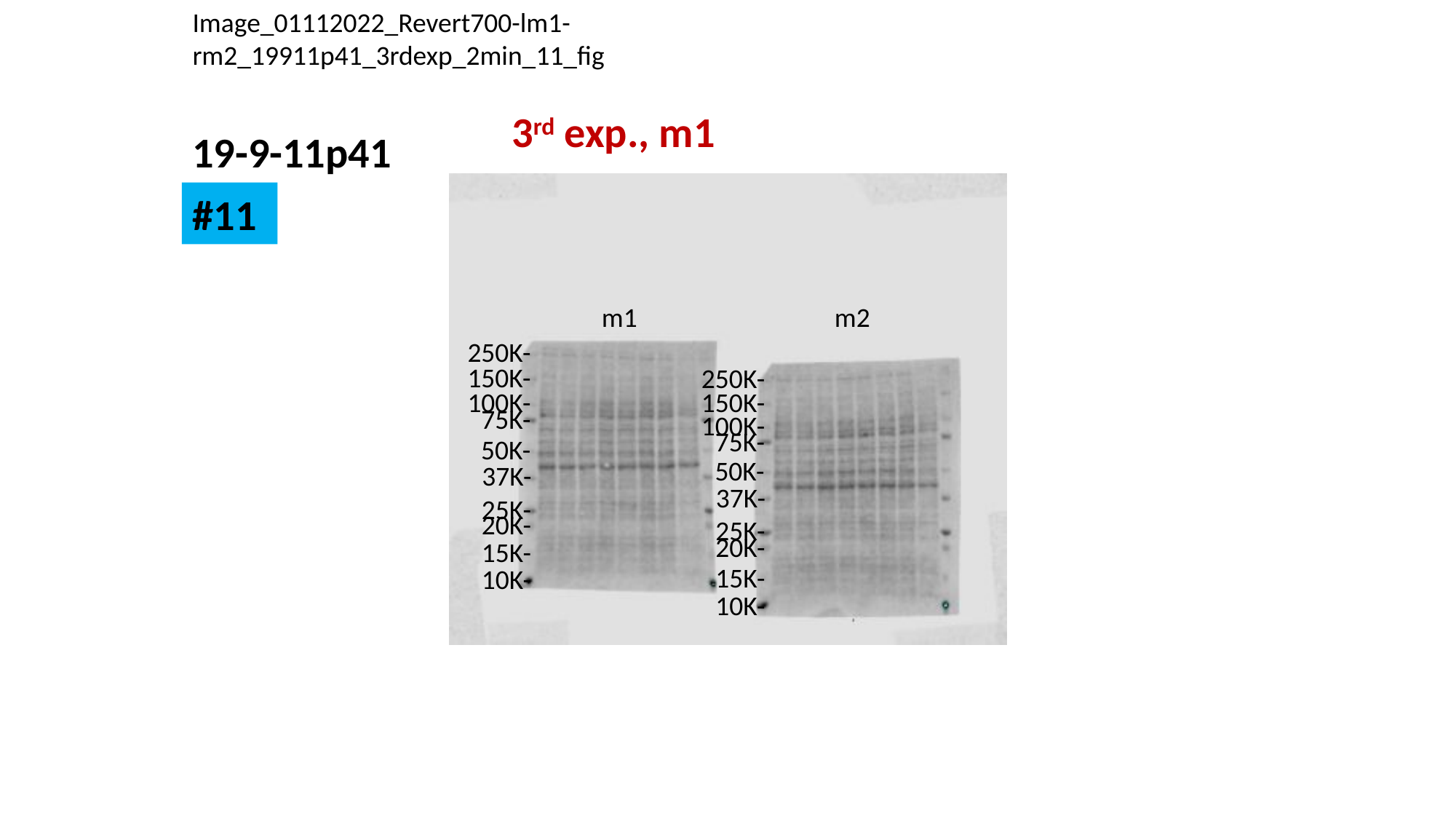

Image_01112022_Revert700-lm1-rm2_19911p41_3rdexp_2min_11_fig
3rd exp., m1
19-9-11p41
#11
m1
m2
250K-
150K-
250K-
150K-
100K-
75K-
100K-
75K-
50K-
50K-
37K-
37K-
25K-
20K-
25K-
20K-
15K-
15K-
10K-
10K-

## Slide 24
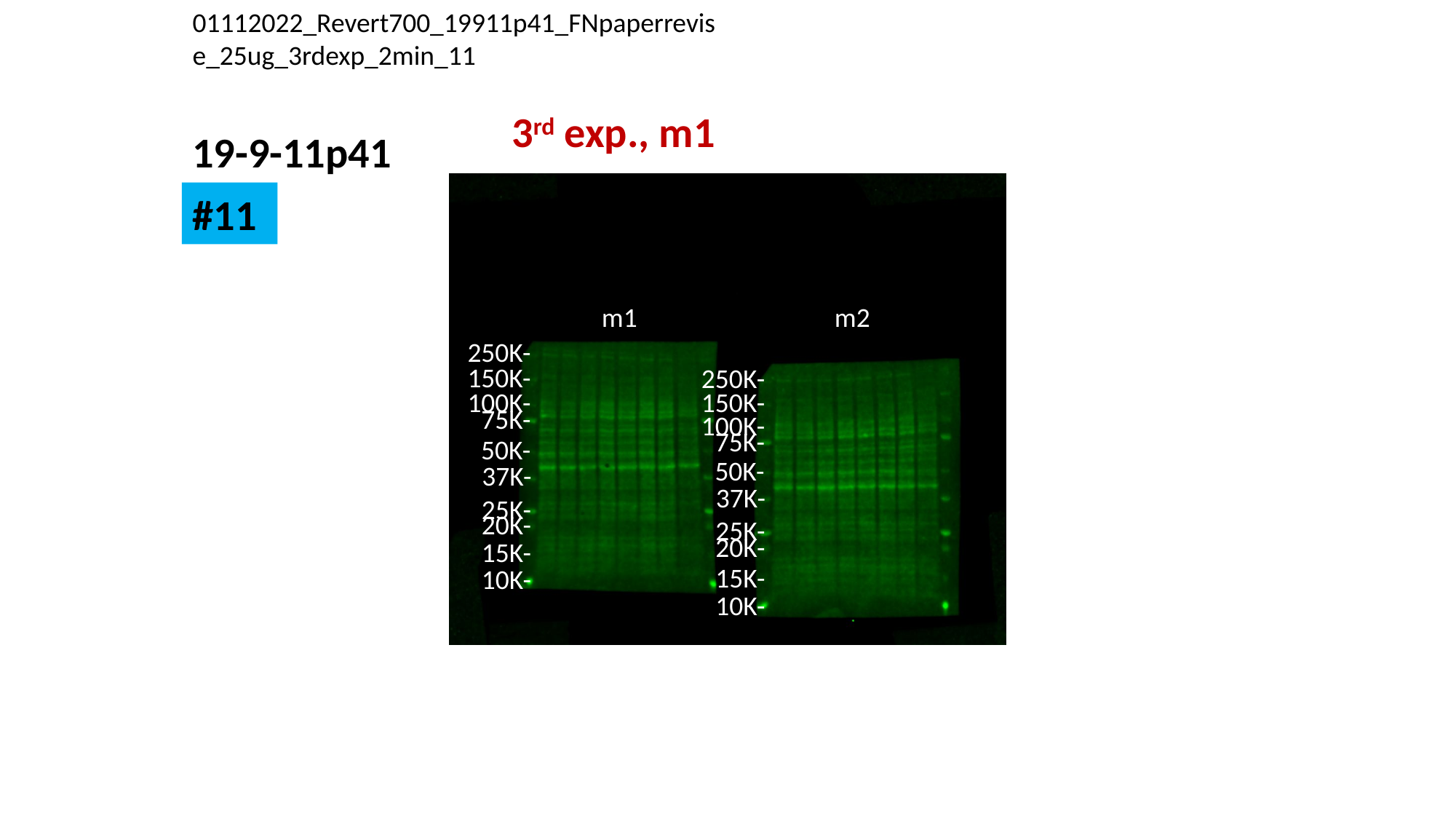

01112022_Revert700_19911p41_FNpaperrevise_25ug_3rdexp_2min_11
3rd exp., m1
19-9-11p41
#11
m1
m2
250K-
150K-
250K-
150K-
100K-
75K-
100K-
75K-
50K-
50K-
37K-
37K-
25K-
20K-
25K-
20K-
15K-
15K-
10K-
10K-
