# Supplementary figures and images for: Cardiac differentiation of human pluripotent stem cells using defined extracellular matrix proteins reveals essential role of fibronectin
Source: eLife. 2022 Jun 27;11:e69028. doi: 10.7554/eLife.69028 (PMC9236614; doi:10.7554/eLife.69028)

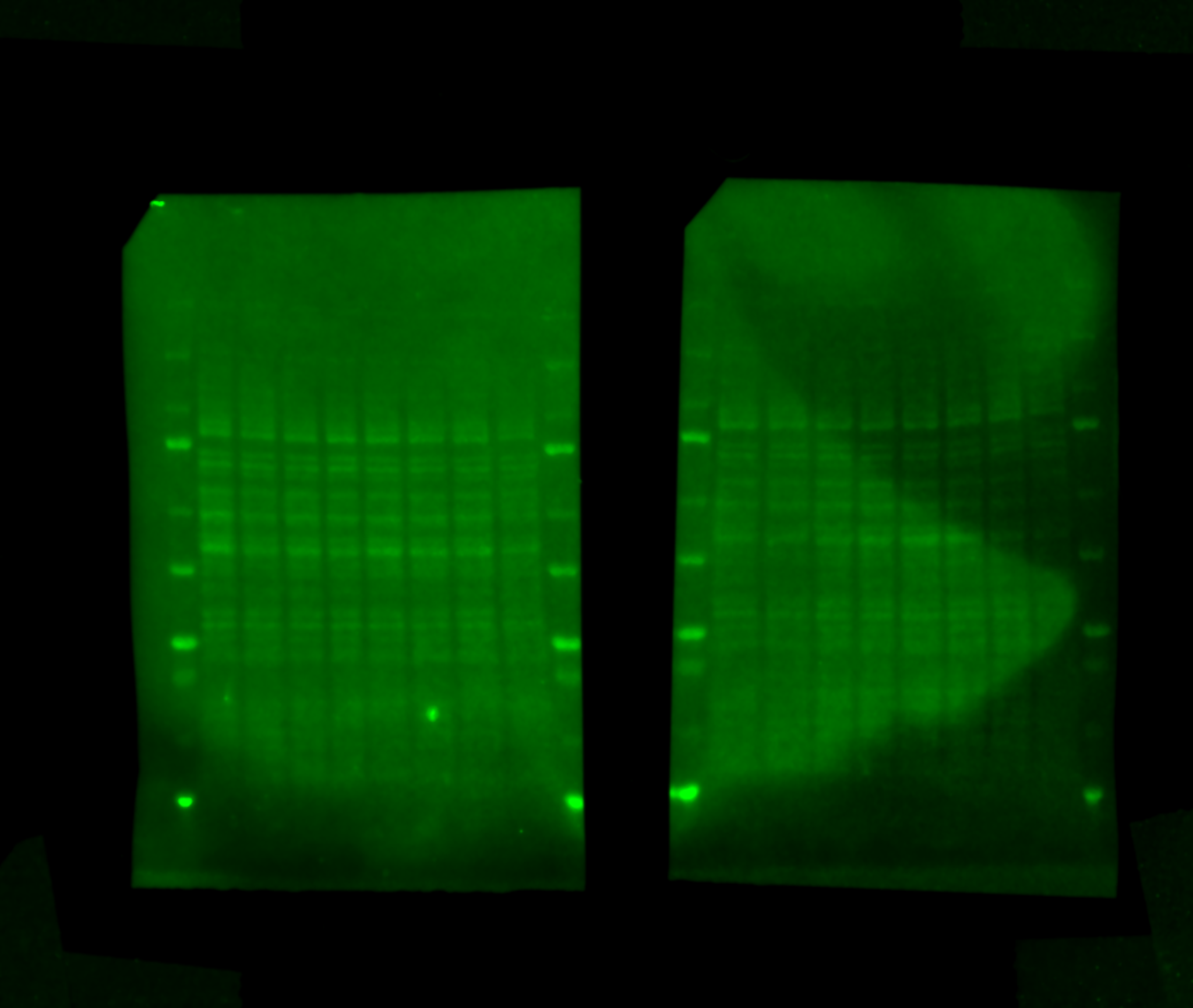

Supplement: Figure 9—source data 1. [file elife-69028-fig9-data1.zip › Figure 9 WB raw data/19-9-11p32/01042022_Revert700-lm1-rm2_19911p32_FNpaperrevise_25ug_1stexp_2min_7.tif]

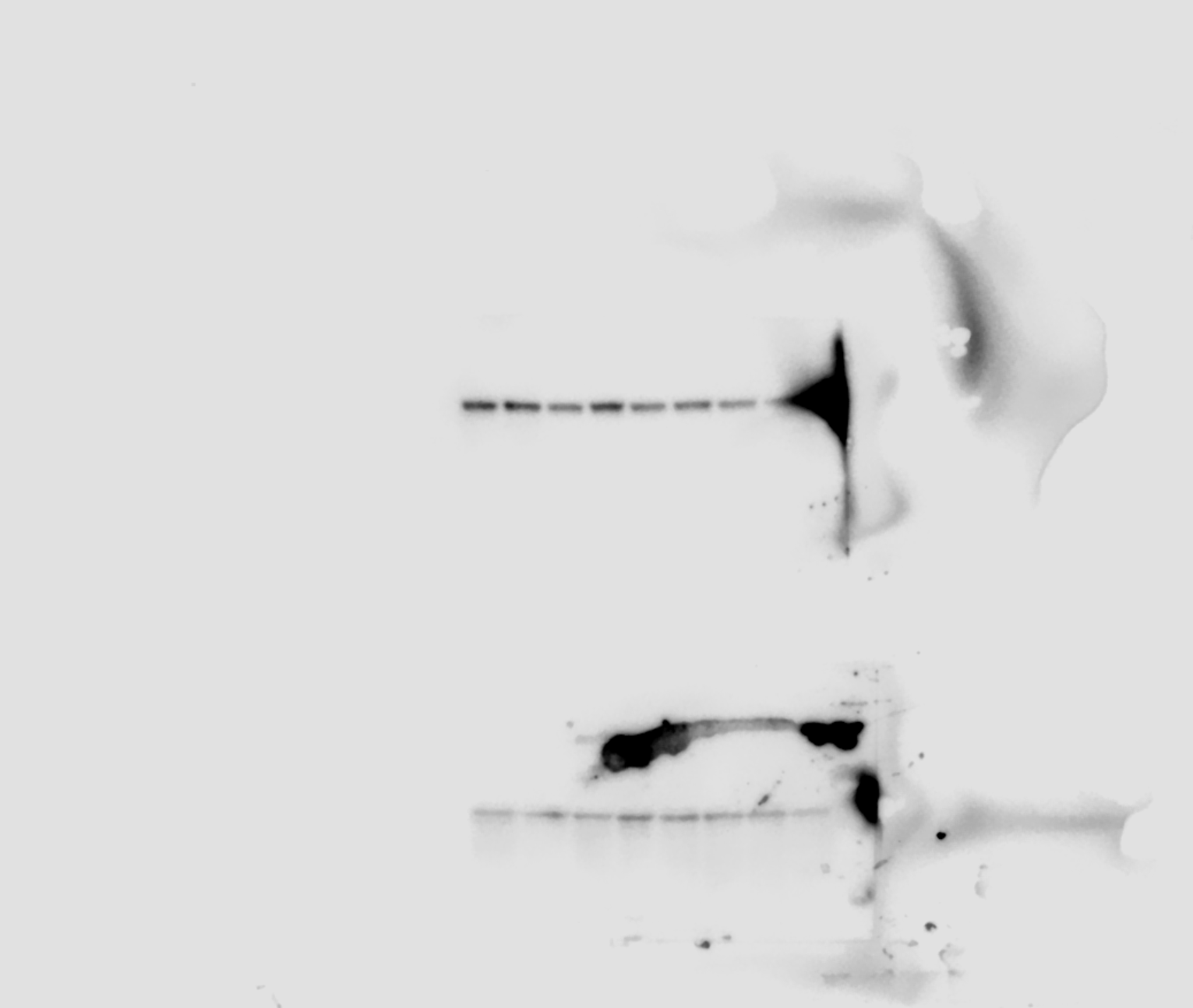

Supplement: Figure 9—source data 1. [file elife-69028-fig9-data1.zip › Figure 9 WB raw data/19-9-11p32/01052022_IB-tpAKTm1-bpGSK3m2_19911p32_FNpaperrevise_25ug_1stexp_10min_12.tif]

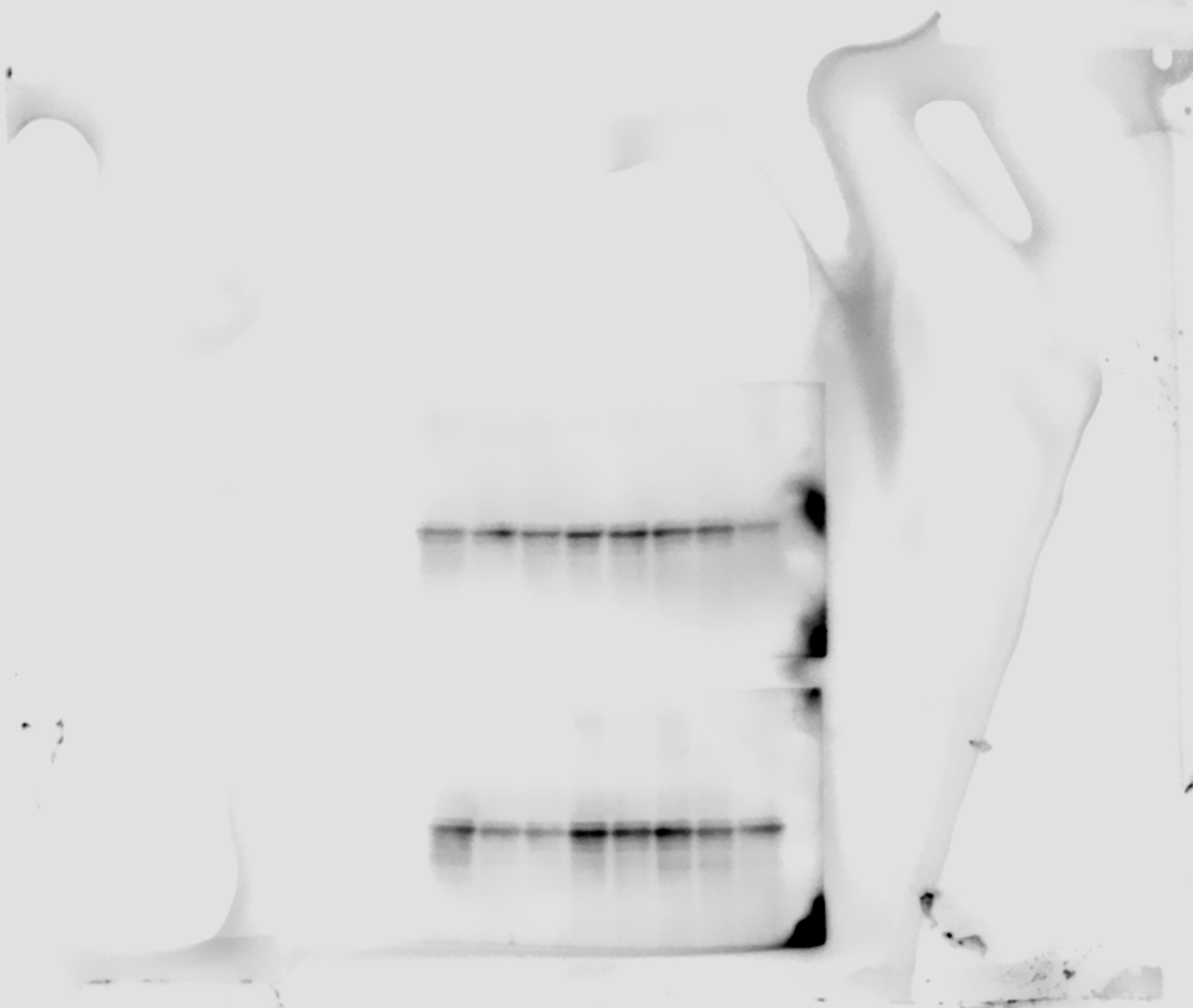

Supplement: Figure 9—source data 1. [file elife-69028-fig9-data1.zip › Figure 9 WB raw data/19-9-11p32/01052022_IB-tpGSK3m2p32-bpGSK3m2p34_FNpaperrevise_25ug_2ndexp_10min_11.tif]

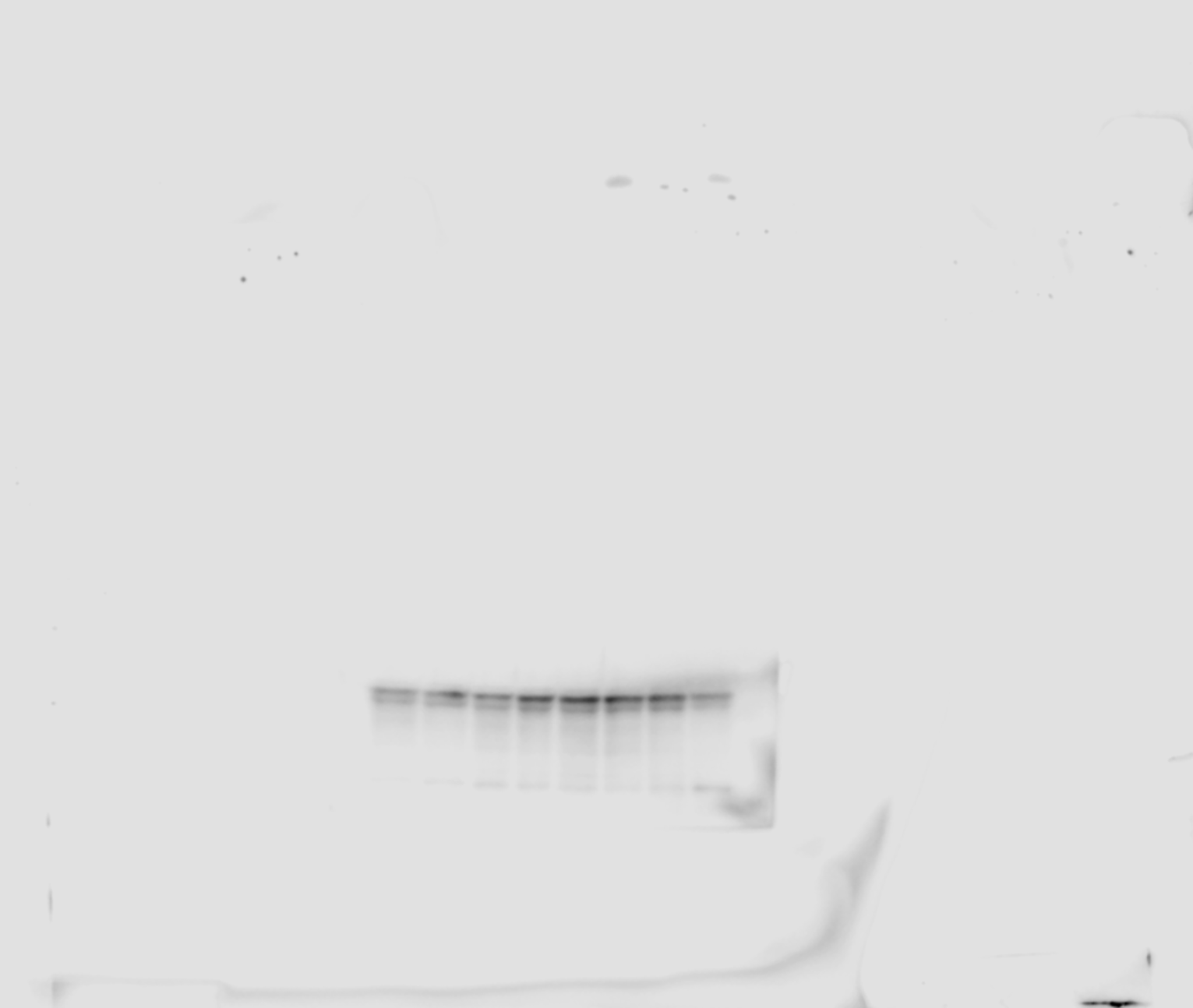

Supplement: Figure 9—source data 1. [file elife-69028-fig9-data1.zip › Figure 9 WB raw data/19-9-11p32/01062022_IB-GSK3m2p32_FNpaperrevise_25ug_2ndexp_10min_12.tif]

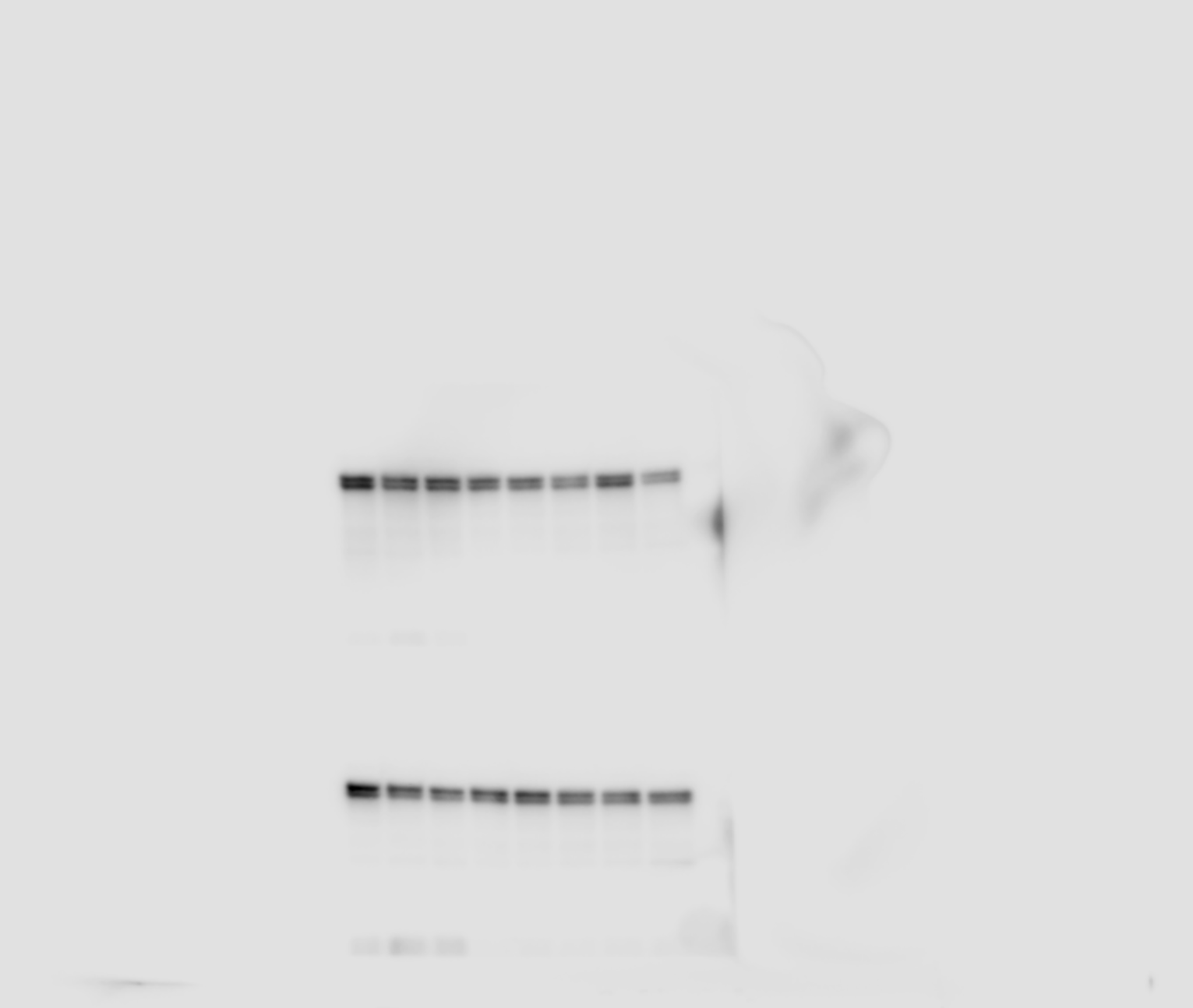

Supplement: Figure 9—source data 1. [file elife-69028-fig9-data1.zip › Figure 9 WB raw data/19-9-11p32/01062022_IB-tAKTm1p32-bAKTm1p34_FNpaperrevise_25ug_1stexp_10min_4.tif]

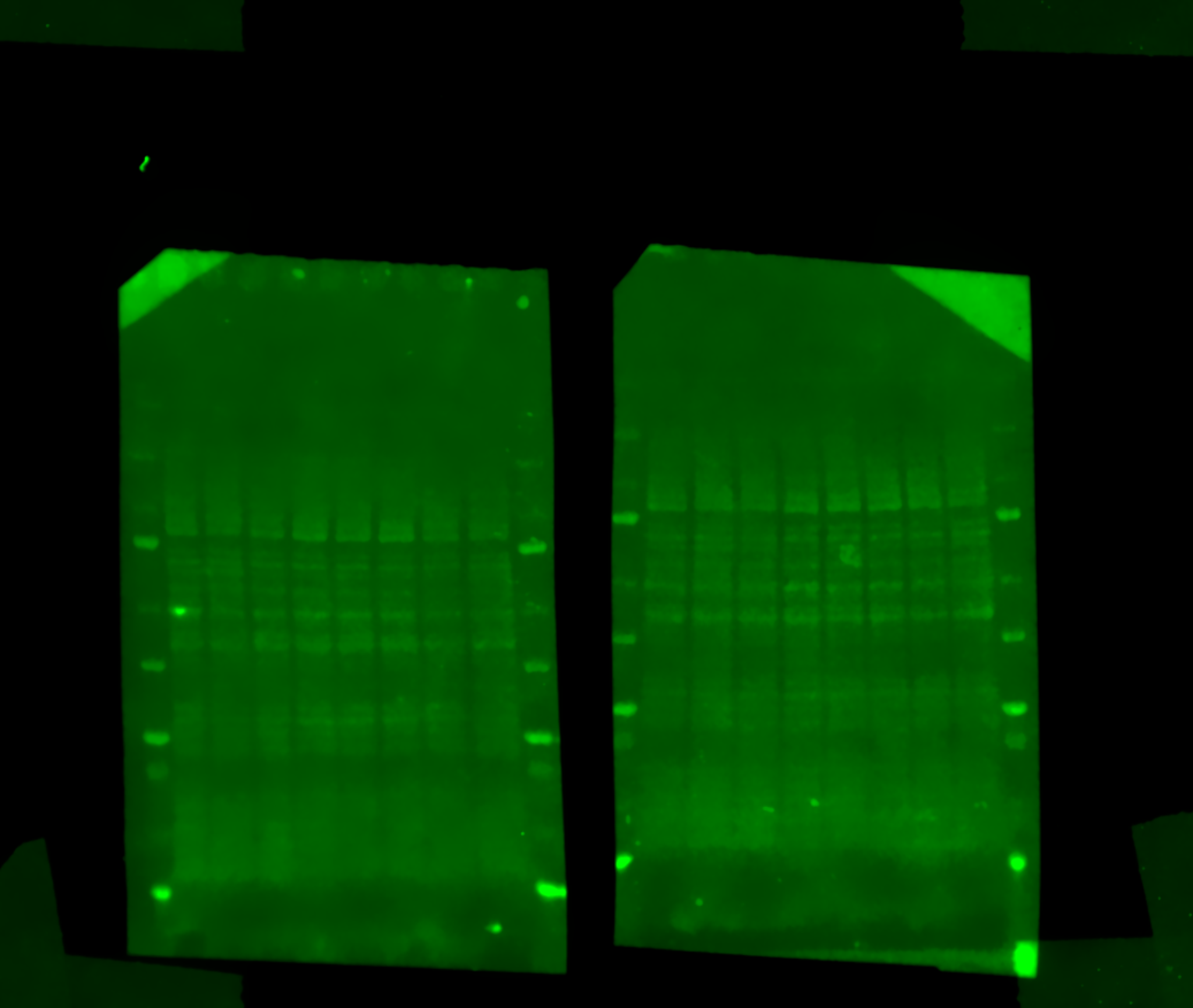

Supplement: Figure 9—source data 1. [file elife-69028-fig9-data1.zip › Figure 9 WB raw data/19-9-11p34/01042022_Revert700-lm1-rm2_19911p34_FNpaperrevise_25ug_1stexp_2min_4.tif]

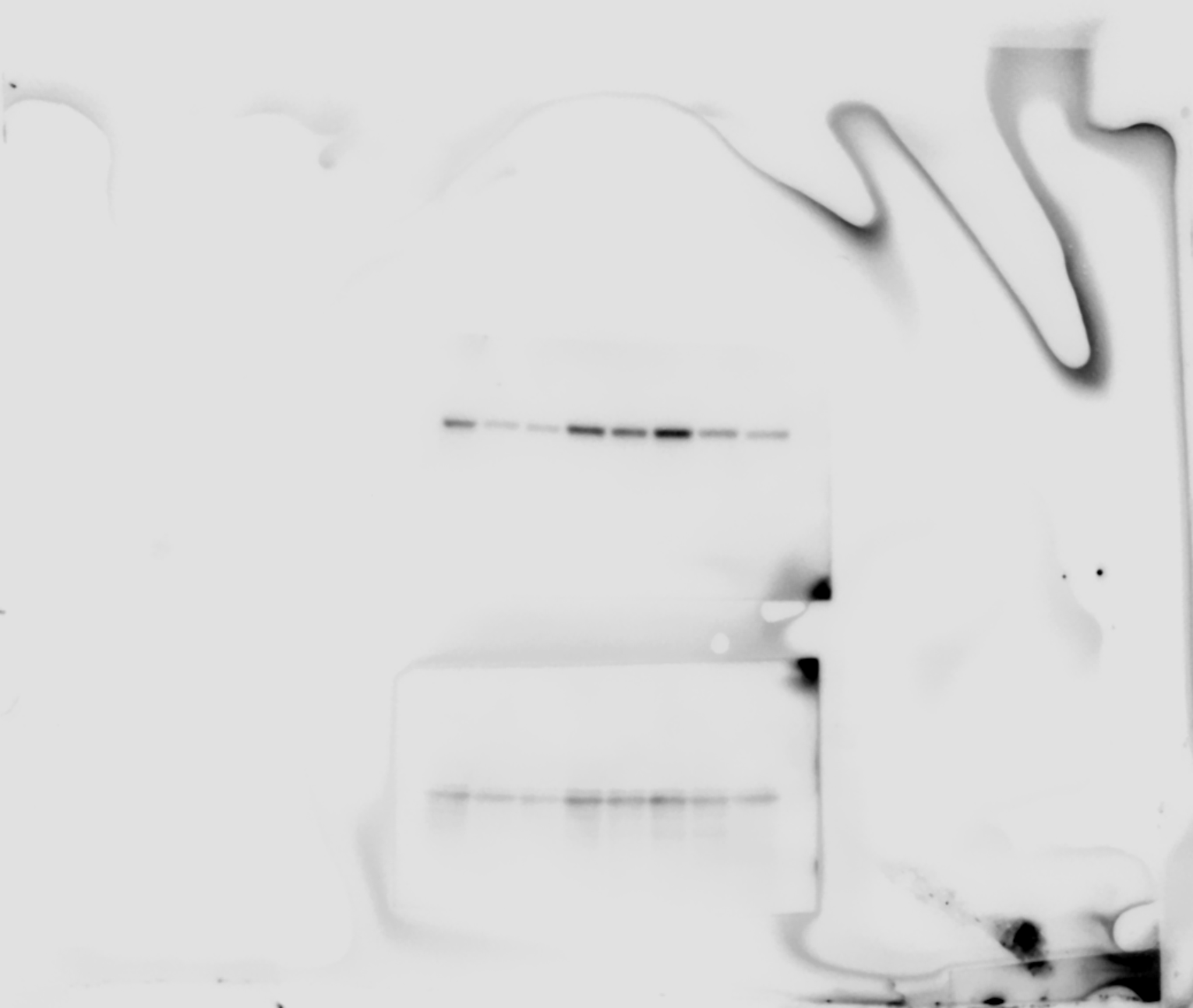

Supplement: Figure 9—source data 1. [file elife-69028-fig9-data1.zip › Figure 9 WB raw data/19-9-11p34/01052022_IB-tpAKTm1-bpGSK3m2_19911p34_FNpaperrevise_25ug_1stexp_10min_5.tif]

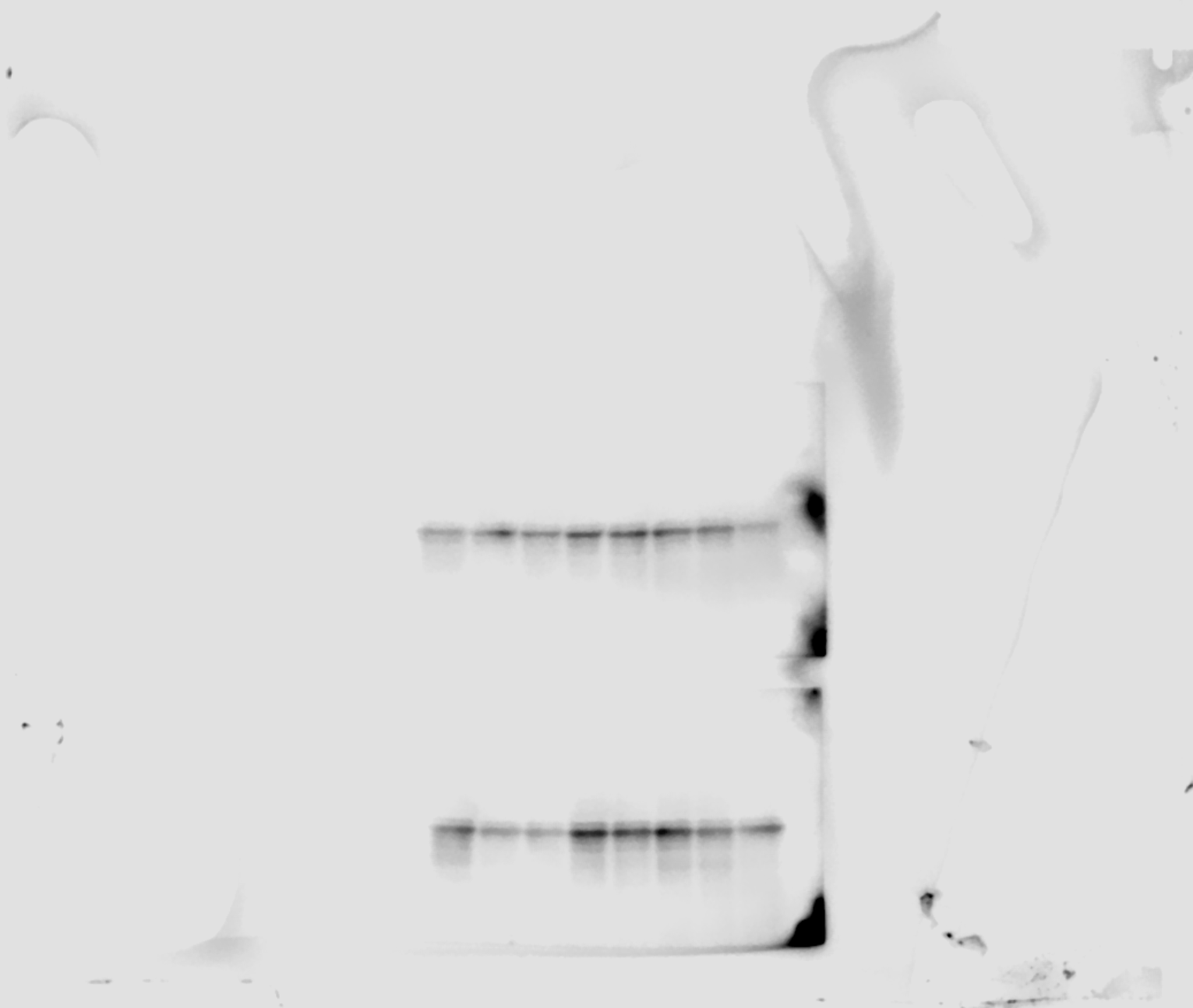

Supplement: Figure 9—source data 1. [file elife-69028-fig9-data1.zip › Figure 9 WB raw data/19-9-11p34/01052022_IB-tpGSK3m2p32-bpGSK3m2p34_FNpaperrevise_25ug_2ndexp_10min_13.tif]

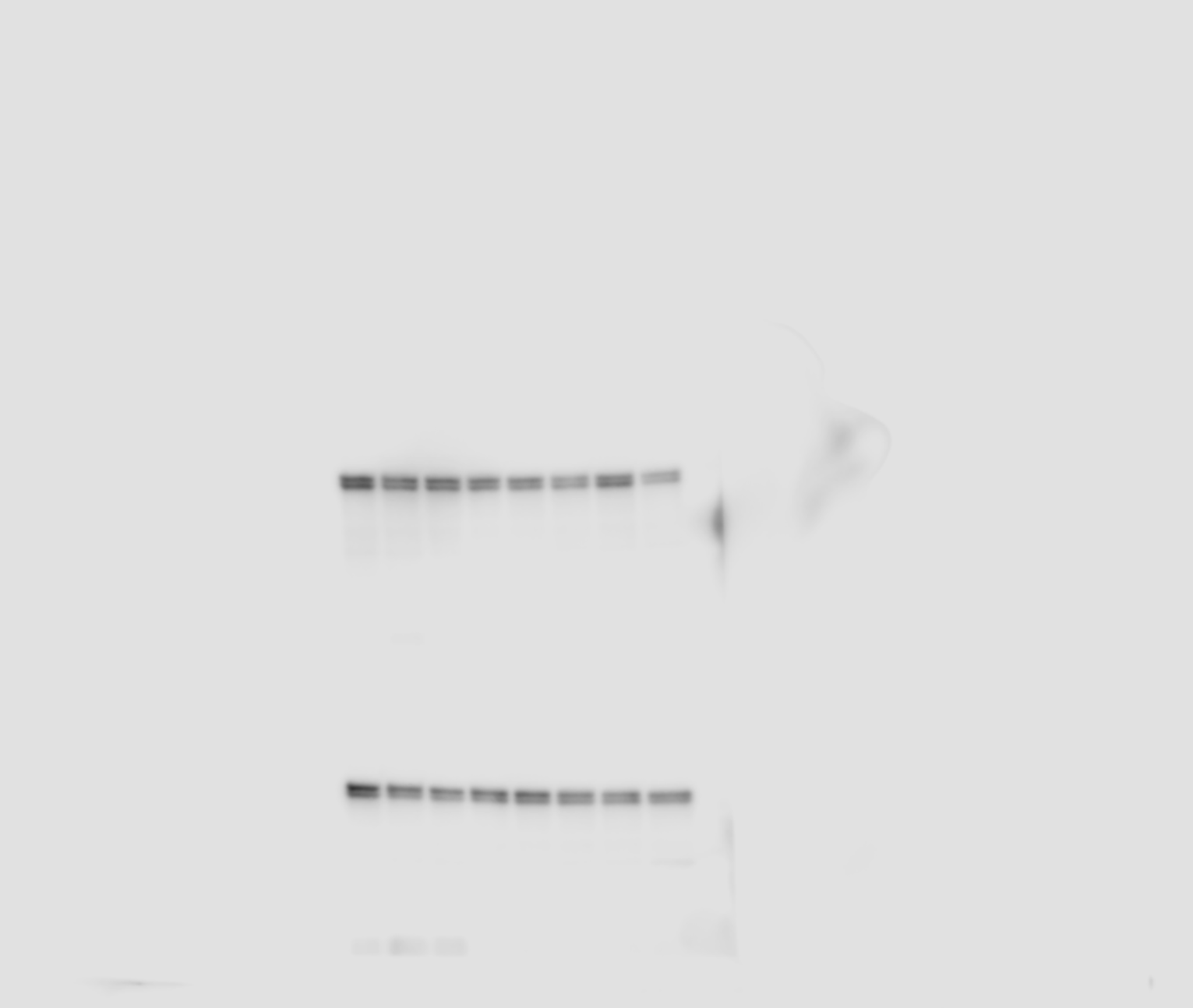

Supplement: Figure 9—source data 1. [file elife-69028-fig9-data1.zip › Figure 9 WB raw data/19-9-11p34/01062022_IB-tAKTm1p32-bAKTm1p34_FNpaperrevise_25ug_1stexp_10min_7.tif]

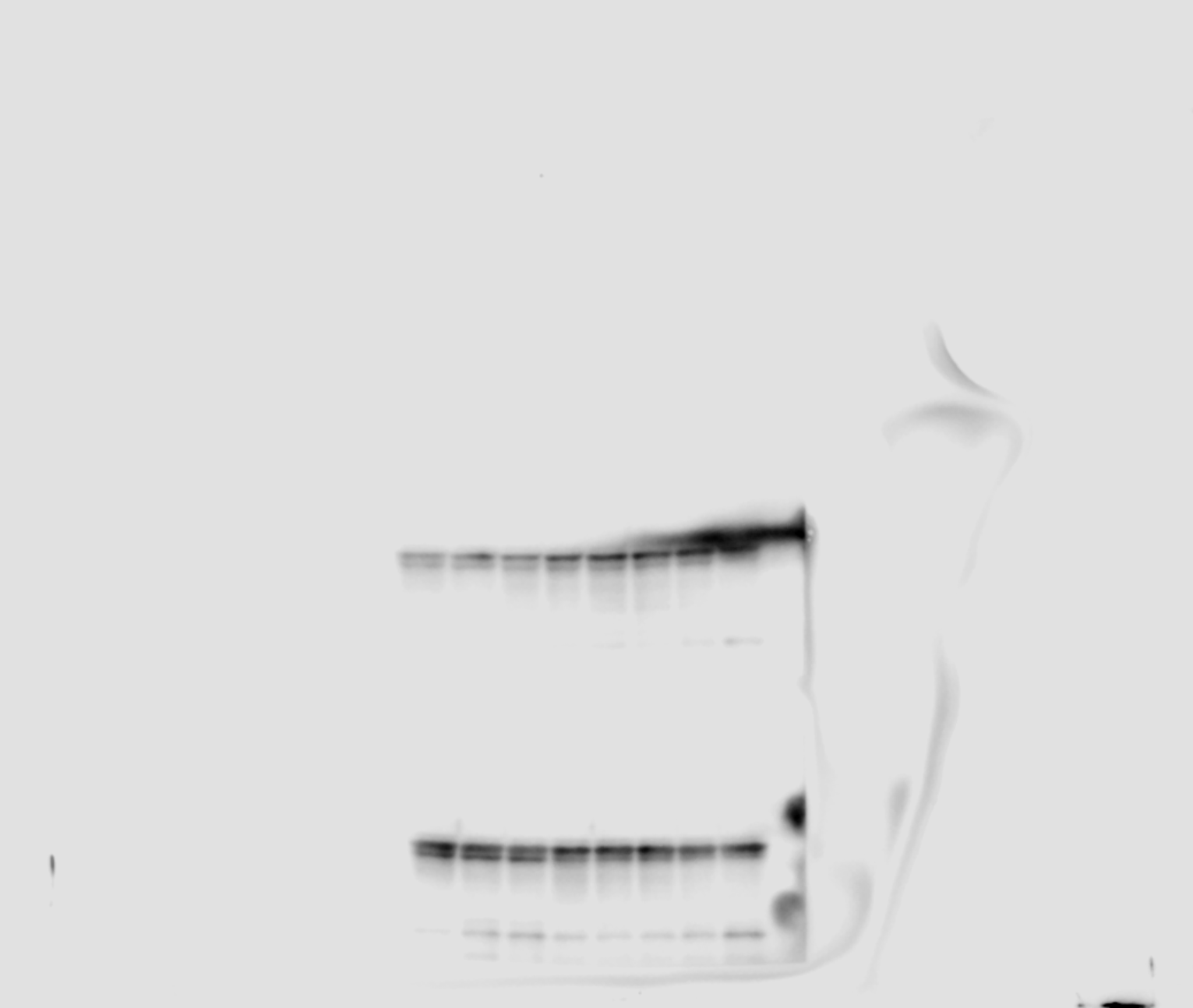

Supplement: Figure 9—source data 1. [file elife-69028-fig9-data1.zip › Figure 9 WB raw data/19-9-11p34/01062022_IB-tGSK3m2p32-bGSK3m2p34_FNpaperrevise_25ug_1stexp_10min_11.tif]

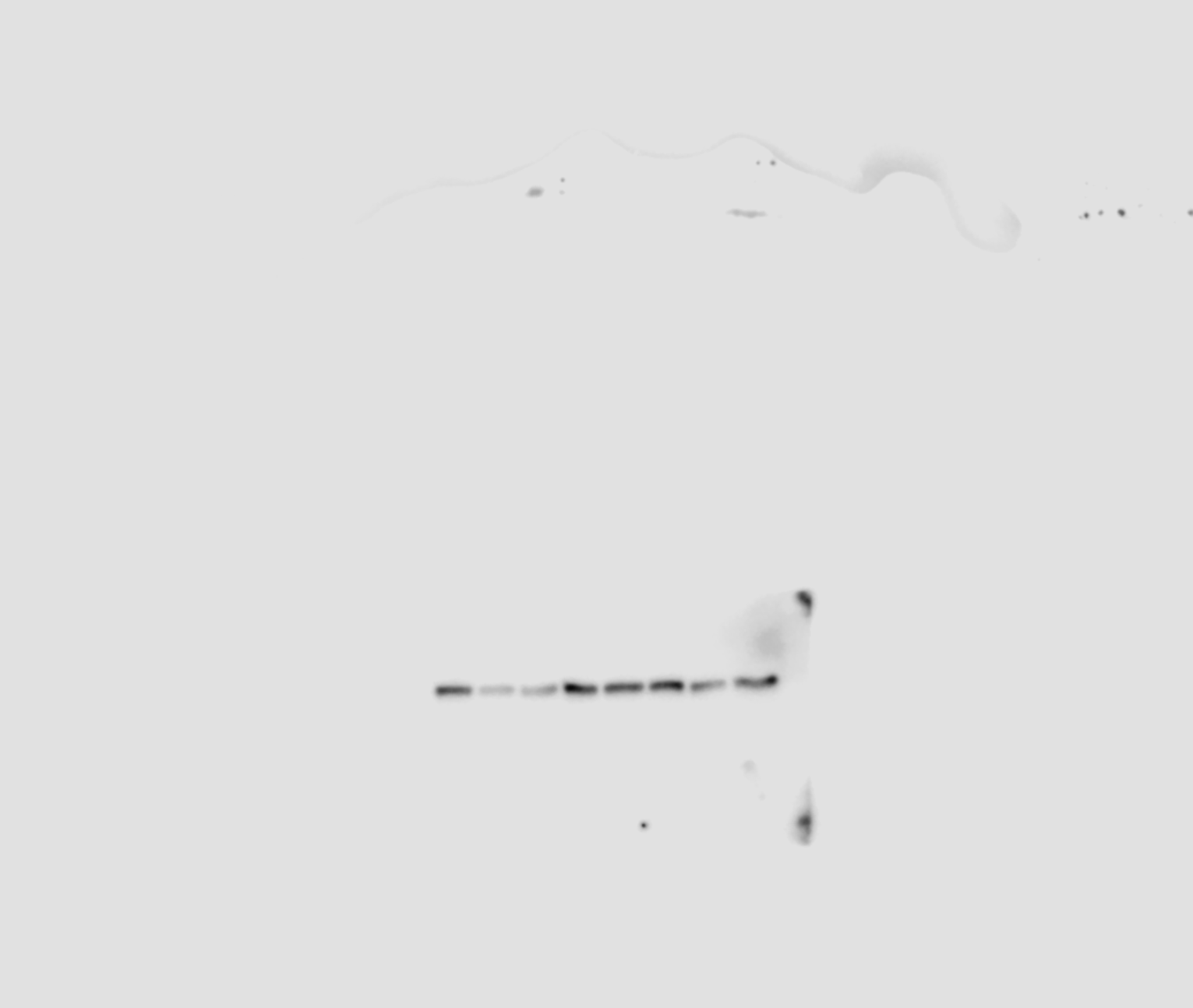

Supplement: Figure 9—source data 1. [file elife-69028-fig9-data1.zip › Figure 9 WB raw data/19-9-11p41/01112022_IB-tpAKT_19911p41_FNpaperrevise_25ug_2ndexp_10min_14.tif]

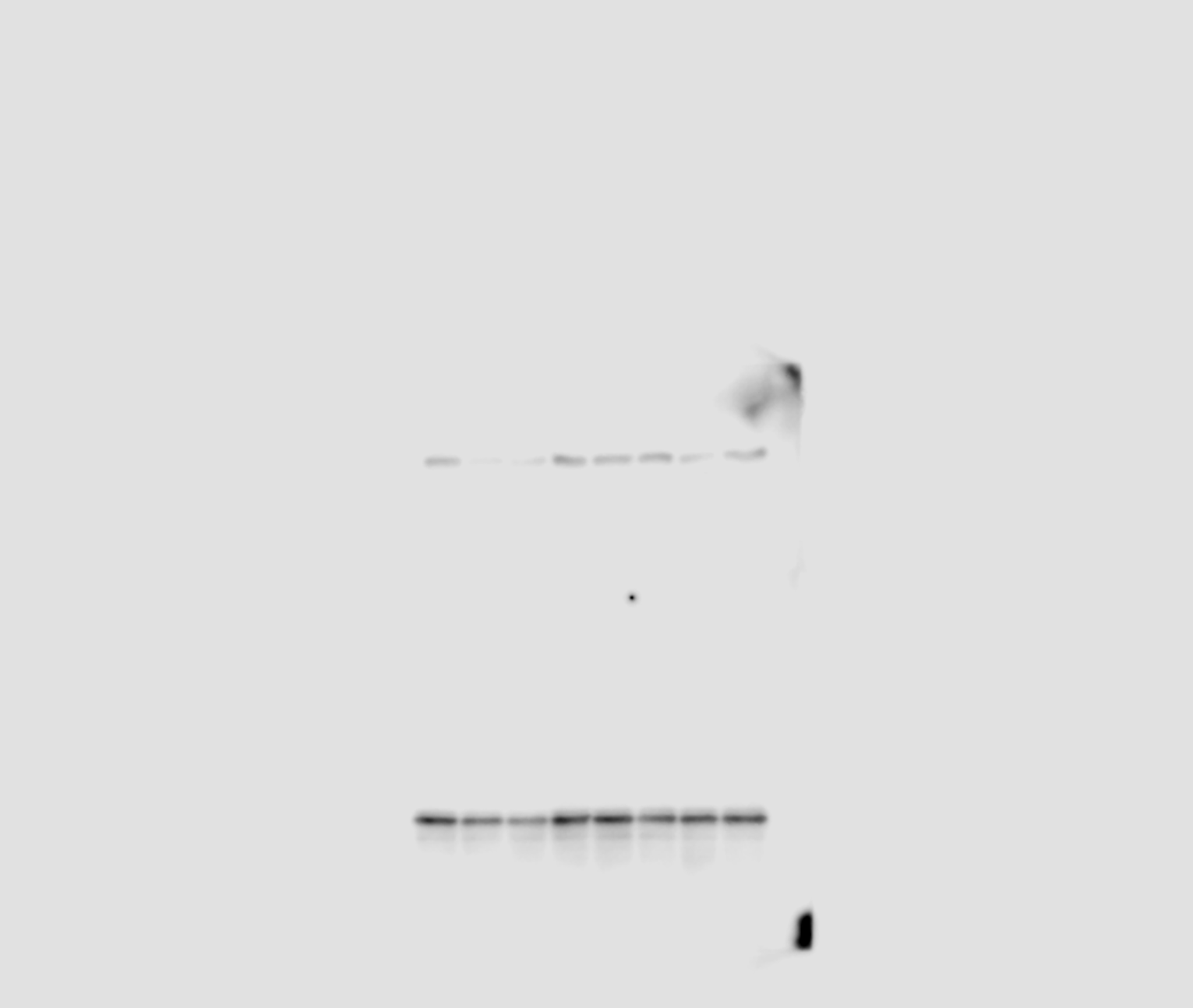

Supplement: Figure 9—source data 1. [file elife-69028-fig9-data1.zip › Figure 9 WB raw data/19-9-11p41/01112022_IB-tpAKT-bpGSK3B_19911p41_FNpaperrevise_25ug_1stexp_10min_16.tif]

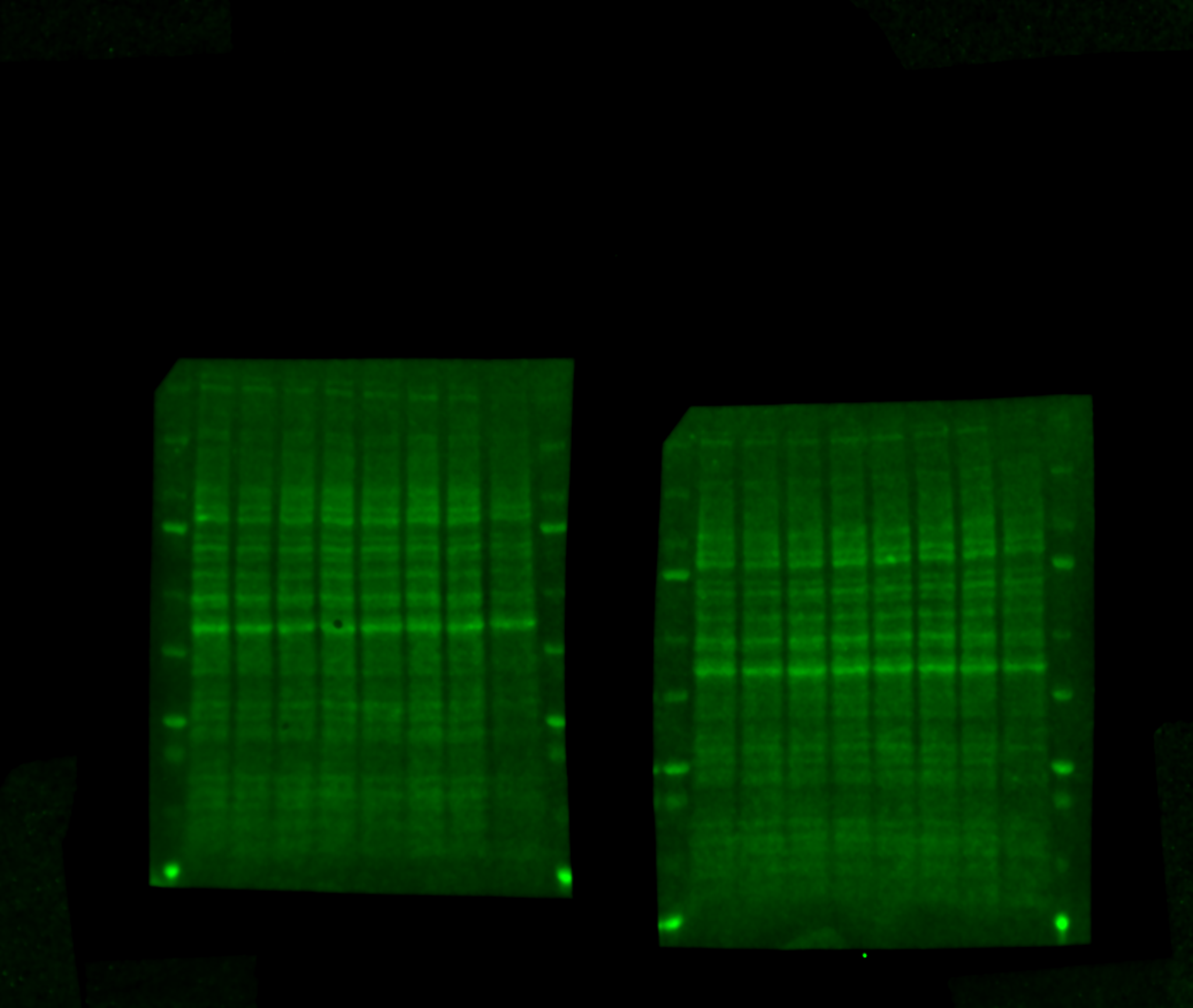

Supplement: Figure 9—source data 1. [file elife-69028-fig9-data1.zip › Figure 9 WB raw data/19-9-11p41/01112022_Revert700_19911p41_FNpaperrevise_25ug_3rdexp_2min_11.tif]

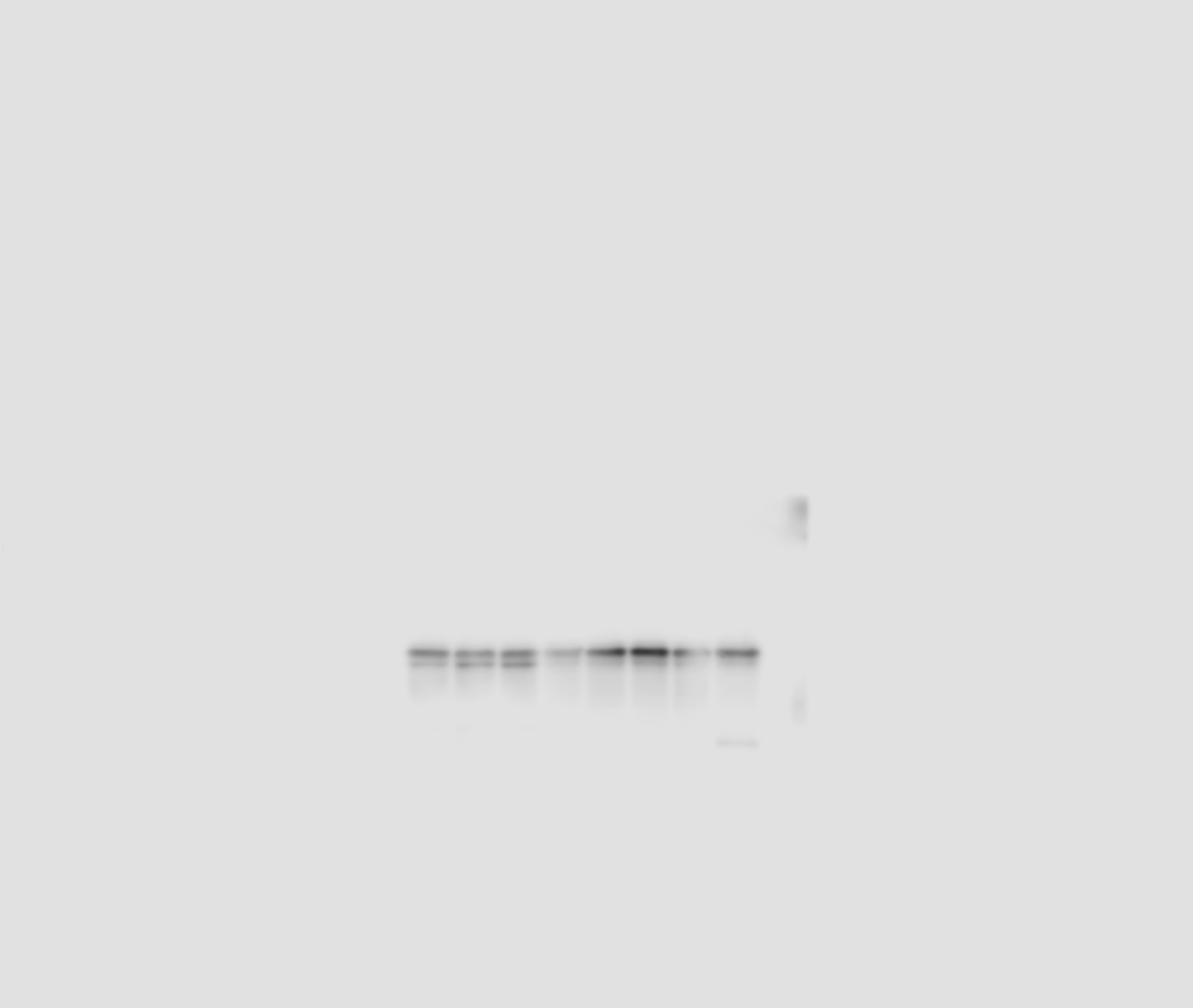

Supplement: Figure 9—source data 1. [file elife-69028-fig9-data1.zip › Figure 9 WB raw data/19-9-11p41/01132022_IB-GSK3B_19911p41_FNpaperrevise_25ug_2ndexp_10min_8.tif]

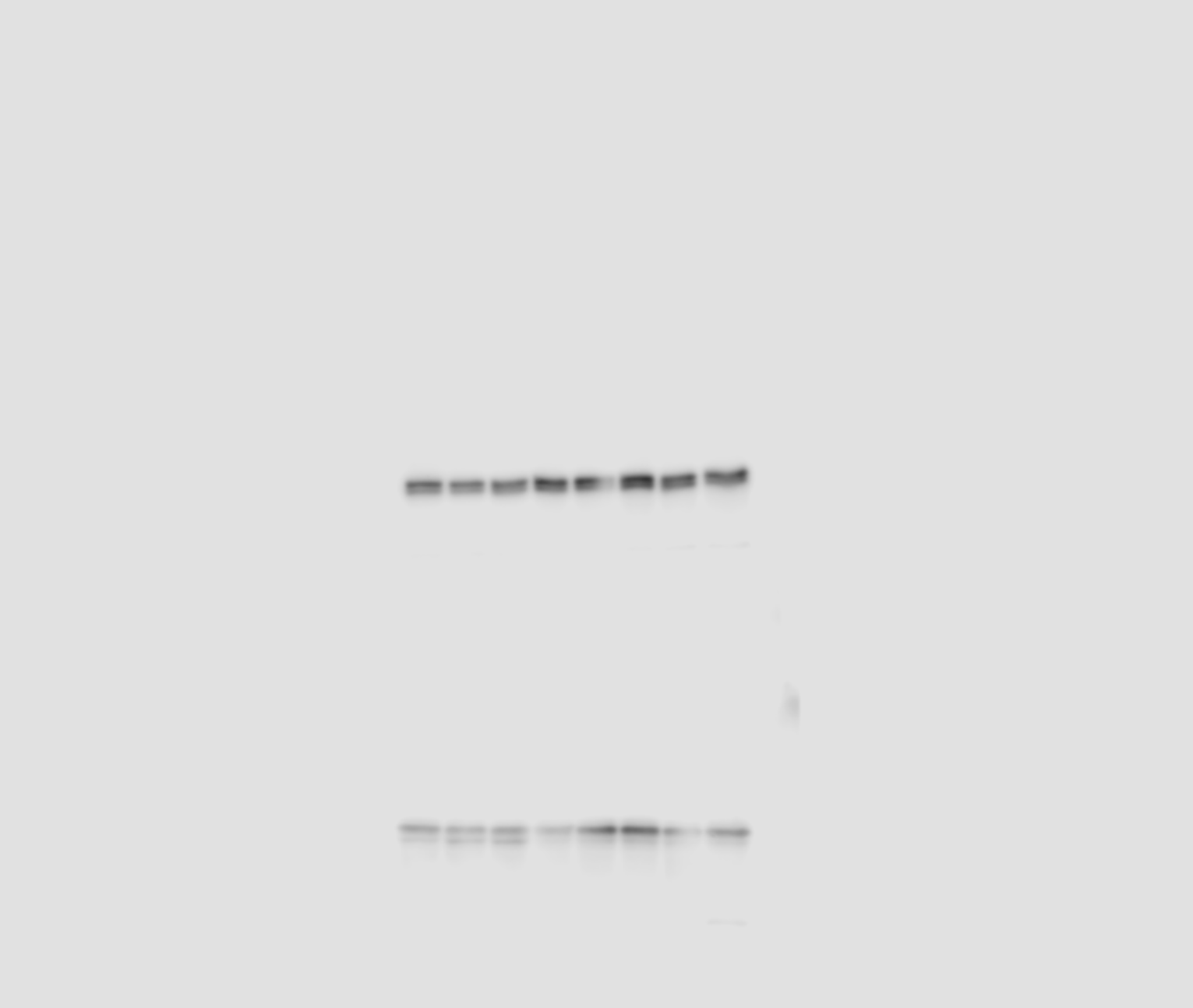

Supplement: Figure 9—source data 1. [file elife-69028-fig9-data1.zip › Figure 9 WB raw data/19-9-11p41/01132022_IB-tAKT-bGSK3B_19911p41_FNpaperrevise_25ug_1stexp_10min_14.tif]
