# Supplementary material for: Cardiac differentiation of human pluripotent stem cells using defined extracellular matrix proteins reveals essential role of fibronectin
Source: eLife. 2022 Jun 27;11:e69028. doi: 10.7554/eLife.69028 (PMC9236614; doi:10.7554/eLife.69028)
Supplement: Supplementary file 1. — (a) Primary antibodies used in immunocytochemistry (ICC) and flow cytometry (FC). (b) Primers for quantitative RT-PCR. (c) Primary antibodies used for immunoblotting. [file elife-69028-supp1.docx]

**Supplementary file 1a.** Primary antibodies used in immunocytochemistry (ICC) and flow cytometry (FC)

| Human/Mouse Brachyury PE-conjugated | R&D (IC2085P) | Goat | IgG | 10ul/1x10^6^ cells (FC) |
| --- | --- | --- | --- | --- |
| Alexa Fluor 647 Mouse Anti-Human Sox17 (Clone P7-969) | BD (562594) | Mouse | IgG_1_ | 5ul/1x10^6^ cells (FC) |
| Human/Mouse Brachyury Antibody | R&D (AF2085) | Goat | IgG | 5ug/ml (ICC) |
| Troponin T, Cardiac Isoform Ab-1 (Clone 13-11) | ThermoFisher Scientific (#MS-295-P) | Mouse | IgG_1_ | 1:200 dilution (FC, ICC) |
| Purified Mouse Anti-Fibronectin (Clone 10) | BD (61007) | Mouse | IgG_1_ | 1:100 dilution (ICC) |
| Anti-Laminin | Sigma-Aldrich (L9393) | Rabbit | IgG | 1:500 dilution (ICC) |
| Oct-3/4 (C-10) | Santa Cruz (sc-5279) | Mouse | IgG_2b_ | 1:100 dilution (ICC) |
| Anti-SSEA4 antibody [MC813-70] | Abcam (ab16287) | Mouse | IgG_3_ | 1:200 dilution (ICC) |

**Supplementary file 1b.** Primers for quantitative RT-PCR

**Genes TaqMan® Gene Expression Assay ID**

GAPDH Hs99999905_m1

SNAIL1 Hs00195591_m1

SNAIL2 Hs00950344_m1

TWIST1 Hs00361186_m1

VIM Hs00185584_m1

FN1 Hs01549976_m1

CDH1 Hs01023894_m1

CDH2 Hs00983056_m1

GSC Hs00418279_m1

MIXL1 Hs00430824_g1

SOX17 Hs00751752_s1

TBXT Hs00610080_m1

MESP1 Hs00251489_m1

ISL1 Hs00158126_m1

NKX2-5 Hs00231763_m1

GATA4 Hs00171403_m1

**Supplementary file 1c.**  Primary antibodies used for immunoblotting

| **Antibody** | **Host/Clonality** | **Vendor** | **Catalog number** | **Lot number** | **Dilution** |
| --- | --- | --- | --- | --- | --- |
| Anti-ILK | Rabbit polyclonal | Cell Signaling Technology | 3862 | 2 | 1:100 |
| Anti-AKT | Rabbit monoclonal | Cell Signaling Technology | 4691 | 28 | 1:500 |
| Anti-AKT-Ser473 | Rabbit polyclonal | Cell Signaling Technology | 9271 | 15 | 1:500 |
| Anti-GSK3β | Rabbit monoclonal | Cell Signaling Technology | 12456 | 10 | 1:500 |
| Anti-GSK3β-Ser9 | Rabbit monoclonal | Cell Signaling Technology | 9323 | 15 | 1:500 |
| HRP linked Anti-Rabbit IgG | Donkey polyclonal | Sigma-Aldrich | GENA934 | 17028694 | 1:1,000 |
| HRP linked Anti-Mouse IgG | Sheep polyclonal | Sigma-Aldrich | GENA931 | 17028693 | 1:1,000 |
